# Comparative Study of Isomeric TFSI and FPFSI Anions in Li-Ion Electrolytes Using Quantum Chemistry and Ab Initio Molecular Dynamics

**DOI:** 10.1021/acs.jpcb.4c08414

**Published:** 2025-02-19

**Authors:** Piotr Kubisiak, Domantas Narkevičius, Chiara Nicotri, Andrzej Eilmes

**Affiliations:** † Faculty of Chemistry, Jagiellonian University, Gronostajowa 2, 30-387 Kraków, Poland; ‡ Faculty of Physics, Vilnius University, 3 Universiteto Street, LT-01513 Vilnius, Lithuania; § Department of Applied Science and Technology, Politecnico di Torino, Corso Duca degli Abruzzi 24, 10129 Torino, Italy

## Abstract

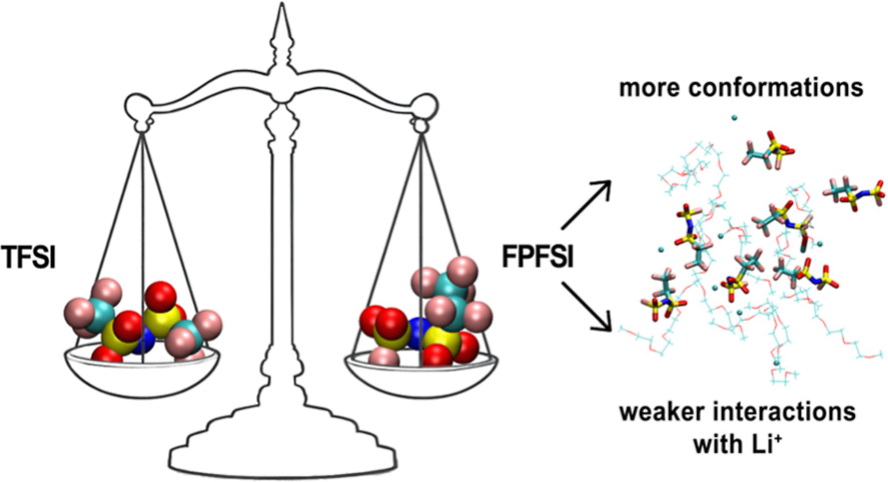

Two isomeric anions used in Li-ion conducting electrolytes, TFSI and FPFSI, have been compared through quantum-chemical calculations. The FPFSI anion has more low-energy conformers, and its asymmetry leads to an increased number of possible structures of FPFSI–Li complexes. The preferred geometry of the anion–Li ion pair for both anions is the bidentate coordination of the cation through two oxygen atoms; the binding effect is slightly weaker for the FPFSI anion. Ab initio molecular dynamics simulations for salt solutions in tetraglyme have revealed that the amount of cation-to-solvent coordination increases in the LiFPFSI electrolytes. Analysis of the vibrational spectra of anions and ion pairs and the IR spectra of electrolytes obtained from the simulations have indicated that the S–F stretching vibration of the FPFSI anion above 600 cm^–1^ can be used in experimental conditions to monitor the FPFSI interactions with lithium cations.

## 1 Introduction

Development of new energy storage devices is of paramount importance for addressing the rising demand in today’s society focused on a sustainable economy. The most successful are the lithium-ion batteries (LIBs), which have been commercially available since the 1990s of the 20th century.^1−4^ The quest for safer, environment-friendly, and more effective LIBs stimulates interest in experimental and theoretical research on energy storage technology.

An ion-conducting electrolyte is an essential component of a battery, contributing to its electrochemical performance. Typical electrolytes for metal ion batteries are salt solutions in molecular solvents or polymer matrices.^5−7^ Commonly applied in commercial devices is lithium hexafluorophosphate (LiPF_6_); however, several more promising salts are investigated experimentally. Among them are salts with weakly coordinating anions, such as lithium bis(trifluoromethanesulfonyl)imide (LiTFSI).^8^ LiTFSI is widely used in research on liquid electrolytes with oligoglyme solvents and solid electrolytes based on poly(ethylene oxide) (PEO); another class of prospective solvents are ionic liquids with TFSI anions. It is therefore not surprising that multiple computational works investigated the properties of TFSI: conformational preferences,^9−11^ binding to lithium cations^12−16^ and the vibrational spectra used to monitor the conformations of the anion and its interactions in an electrolyte.^9,10,13,14,16^ Molecular dynamics (MD) simulations were employed to study the structure and dynamics of LiTFSI solutions in molecular^17−20^ or ionic liquids.^21−23^

Recently, several asymmetric perfluorinated sulfonimide anions were studied experimentally,^24−27^ including the TFSI isomer, (fluorosulfonyl)(pentafluoroethanesulfonyl)imide (FPFSI).^24,26,27^ The LiFPFSI/PEO electrolytes exhibited relatively high conductivities and improved stability, making LiFPFSI a promising salt for future works.^26^ While the symmetric TFSI anion was theoretically studied in a vast number of works, not much modeling has been performed on its asymmetric counterpart, FPFSI. The density functional theory (DFT) based calculations were reported for a series of perfluorinated anions and their complexes with Li^+^.^28^ The general conclusions of ref (28) were that asymmetric anions show enhanced flexibility of the S–N bond and lower interaction energies with Li cations. Nevertheless, only a few selected geometries of Li–anion pairs were examined. The calculations were performed using an implicit continuous solvent, therefore the effect of explicit solvation on the structure of ion complexes is not obvious. In a more recent work,^29^ DFT was used for screening of a set of lithium salts, including LiFPFSI, but only the lowest-energy structures of ion pairs in vacuum were analyzed. Neither of these works^28,29^ discussed the effects of Li–anion interactions on vibrational spectra.

The computational comparison of two isomeric anions and the relation of their structure to the properties is an interesting case, therefore we decided to perform a more detailed study of FPFSI anions and LiFPFSI-based electrolytes. To that end, we used quantum chemical calculations to find the conformations of free anions, the geometries and binding energies of their complexes with Li^+^, and the infrared (IR) or Raman spectra of low-energy structures. Next, we employ ab initio molecular dynamics (AIMD) simulations of salt solutions in tetraglyme (G4) to confront the behavior of a condensed phase system with the predictions made for isolated ions or aggregates. G4 was chosen as the solvent, because it was used in several experimental or computational studies on LiTFSI solutions.^16,17,19,20,30^ Although the TFSI anion and LiTFSI complexes have already been well studied in the literature, we repeat the calculations for these systems to compare the data for both isomers obtained using exactly the same methodology.

## 2 Computational Methods

The structures of both anions and the labeling of the atoms are shown in Figure S1 in the Supporting Information. Gaussian 09 rev. D.01 software^31^ was used for quantum-chemical (QC) calculations. We applied the ab initio MP2 method^32^ and the DFT methodology^33,34^ with three functionals: PBE^35^ (Generalized Gradient Approximation functional commonly used in solid-state physics), B3LYP^36^ (one of the most widely used hybrid functionals), and the M062X hybrid.^37^ In all cases the aug-cc-pVDZ basis set was used. The empirical dispersion correction D3^38^ was added to the potential energy in the DFT calculations. The MP2 results, free from the arbitrariness of the choice of the exchange-correlation functional, will serve as a kind of reference. The computational cost of MP2 calculations becomes prohibitive for larger systems and for AIMD simulations, therefore one expects that DFT methods will be used in these cases. Here, we want to compare two popular hybrid functionals. Nevertheless, hybrid DFT functionals still are quite expensive in AIMD and therefore we also investigate the PBE, significantly cheaper and widely used in the studies of condensed phase.

Multiple initial geometries, based on preliminary scans of dihedral angles, were used for isolated anions in order to locate different local minima of potential energy. Accordingly, for ion pairs, several starting structures were prepared by placing Li^+^ in different positions around a few chosen conformations of the anion. Harmonic frequency calculations followed geometry optimizations to verify the nature of the stationary point; for the lowest energy structures IR and Raman spectra were calculated in the harmonic approximation. The default SCF convergence criteria (energy change ≤10^–6^ a.u., RMS change of the density matrix ≤10^–8^ a.u.) and tight geometry optimization convergence (maximum force ≤0.000015 a.u., RMS force ≤0.00001 a.u., maximum displacement ≤0.00006 a.u., RMS displacement ≤0.00004 a.u.) were used. The ultrafine integration grid was requested in DFT calculations. Integral equation formalism of the polarizable continuum model (PCM) with default settings (Universal Force Field radii and scaled van der Waals surface used to construct the cavity) were applied in the implicit solvent calculations. The value of the static dielectric constant of the solvent was set to 5.

Ab initio molecular dynamics simulations were performed for electrolytes with LiTFSI or LiFPFSI salt dissolved in tetraglyme. Two salt concentrations were examined, corresponding to Li/O(glyme) ratios 1:20 and 1:8, as used in the experimental work.^26^ With the limited size of the systems tractable by AIMD, the number of Li–anion pairs in these structures was 3 and 7, respectively; detailed compositions of electrolytes are given in Table S1 in the Supporting Information. All systems were constructed using Packmol software.^39^ Two series of initial structures were used: in set a, Li^+^ cations were introduced in the form of Li–G4 complexes, in set b, cations were placed independently. Initial equilibration through classical molecular dynamics was performed in the *NVT* ensemble (*T* = 303 K) using the Tinker 5.1 package^40^ and the force field adapted from the APPLE&P parametrization.^41^ The size of periodic simulation boxes was set to reproduce the experimental densities of LiTFSI/G4 electrolytes;^30^ the same densities were assumed for the LiFPFSI/G4 systems. For the systems of type *a*, rigid-body dynamics was applied, therefore the coordination of the Li^+^ cations to G4 molecules was preserved. Standard (with flexible molecules) *NVT* simulations were performed for systems *b*. Therefore, after the 25 ns of equilibration in classical MD simulations, all cations in systems *a* were coordinated solely to the solvent molecules, whereas in systems *b*, Li^+^ ions also interacted with salt anions.

Initially equilibrated structures were then used as starting points for AIMD simulations in the CP2K package.^42,43^ The PBE functional was used, with the D3 dispersion correction, Goedecker’s pseudopotentials,^44^ and a molecularly optimized DZVP-MOLOPT-GTH basis set.^45^ The AIMD simulations were performed for 50 ps in the *NVT* ensemble at *T* = 303 K with a time step of 1.0 fs. An additional simulation was performed for neat G4 liquid.

The IR spectra were computed from the AIMD trajectories as the Fourier transform (FT) of the autocorrelation function of the total dipole moment of the system. Additionally, power spectra of local vibrations were obtained as FTs of selected bond lengths. For clear presentation, the calculated frequencies were convoluted with Gaussian curves with σ = 10 cm^–1^ (IR spectra) or σ = 20 cm^–1^ (power spectra). We also calculated the power spectra as FTs of the velocity autocorrelation function using Travis software.^46^

## 3 Results and Discussion

### 3.1 Structure and Binding Energies

**Figure 1:**
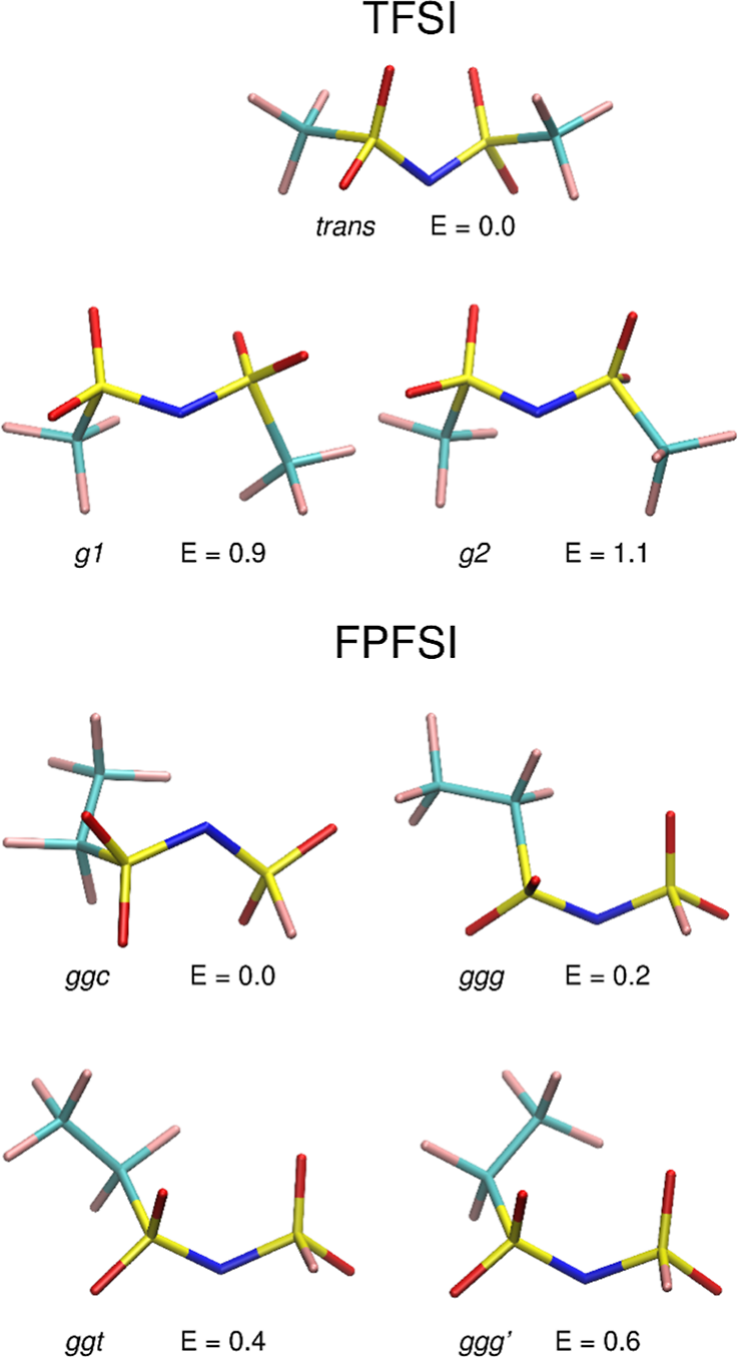
Lowest-energy conformations of TFSI and FPFSI anions calculated at the MP2/aug-cc-pVDZ level in vacuum. Relative energies in kcal/mol.

Geometries of the few lowest energy conformations of both ions, calculated in vacuum within the MP2 methodology are shown in Figure 1 along with their energies relative to the lowest conformer of a given anion. As already well-known from several studies,^9,12^ the most stable conformation of the TFSI anion is the trans geometry, followed by the gauche conformation at energy about 1 kcal/mol higher. We found two gauche structures, denoted here as *g1* and *g2*, differing in the values of the CSSC dihedral angle: 39.9 and 76.7°, accordingly. There are some other structures (cis and gauche conformations) at higher energies, but corresponding to saddle points of the potential energy surface; energies and torsional angles for these structures are listed in Table S2 in the Supporting Information.

The presence of two gauche structures is somewhat surprising, because earlier works reported only one gauche conformation of the TFSI anion.^9,12,47^ We verified that both our structures are the minima of the potential energy surface (no imaginary frequencies) for all methods (MP2 and DFT, vacuum or PCM) used in calculations in this work. Cartesian coordinates for the final structures obtained at the MP2/aug-cc-pVDZ in vacuum are provided in the Supporting Information. Closer inspection of the data from the literature revealed that both conformations have already been described, and the results depend on the computational method used. Values of the CSNS dihedral angles listed in ref (47) for the C_1_ structure are close to those found for our *g2* conformer. On the other hand, a recent work^48^ reports dihedral angles corresponding to the *g1* geometry. In table 1 of ref (12), CSSC and CSNS angles of the C_1_ structure obtained in the Hartree–Fock method correspond to the *g2* geometry, while the B3LYP calculations yielded the *g1* structure. It is therefore possible that for our combinations of the computational method and the basis set (aug-cc-pVDZ), both conformations are the minima in the relatively flat region of the potential energy surface, but the change in the computational methodology (e.g., an increase of the basis set size) would result of a disappearance of the higher minimum. Nevertheless, in the calculations reported here, there are two gauche geometries of the TFSI anion; therefore, we presented both of them in the analysis. We should note that this issue of two conformers at very close energies found for an isolated anion is of rather limited relevance to the real electrolyte, where the preferred conformations of anions result mainly from interactions with cations and/or the solvent.

The potential energy of the FPFSI ion is approximately 8.6 kcal/mol lower than that for the trans conformation of TFSI, suggesting that the former isomer is more stable thermodynamically. Several different orientations of the C_2_F_5_ tail with respect to the core of the FPFSI anion, together with the asymmetry of the SO_2_F group, lead to an increasing number of possible structures. The four lowest geometries, with relative energies up to 0.6 kcal/mol, are displayed in Figure 1 and labeled *ggc*, *ggg*, *ggt*, and *ggg*′, according to the conformation (cis, gauche or trans) at three dihedral angles, FSNS, SNCC and NSCC. Structural data are collected in Table S3. In the lowest-energy conformers, the FSNS and SNCC dihedral angles correspond to gauche conformations, with the former being close to 76° and the latter changing between 75 and 100°. The value of the NSCC dihedral, changing the orientation of the C_2_F_5_ group, has only a minor effect on the potential energy.

**Figure 2:**
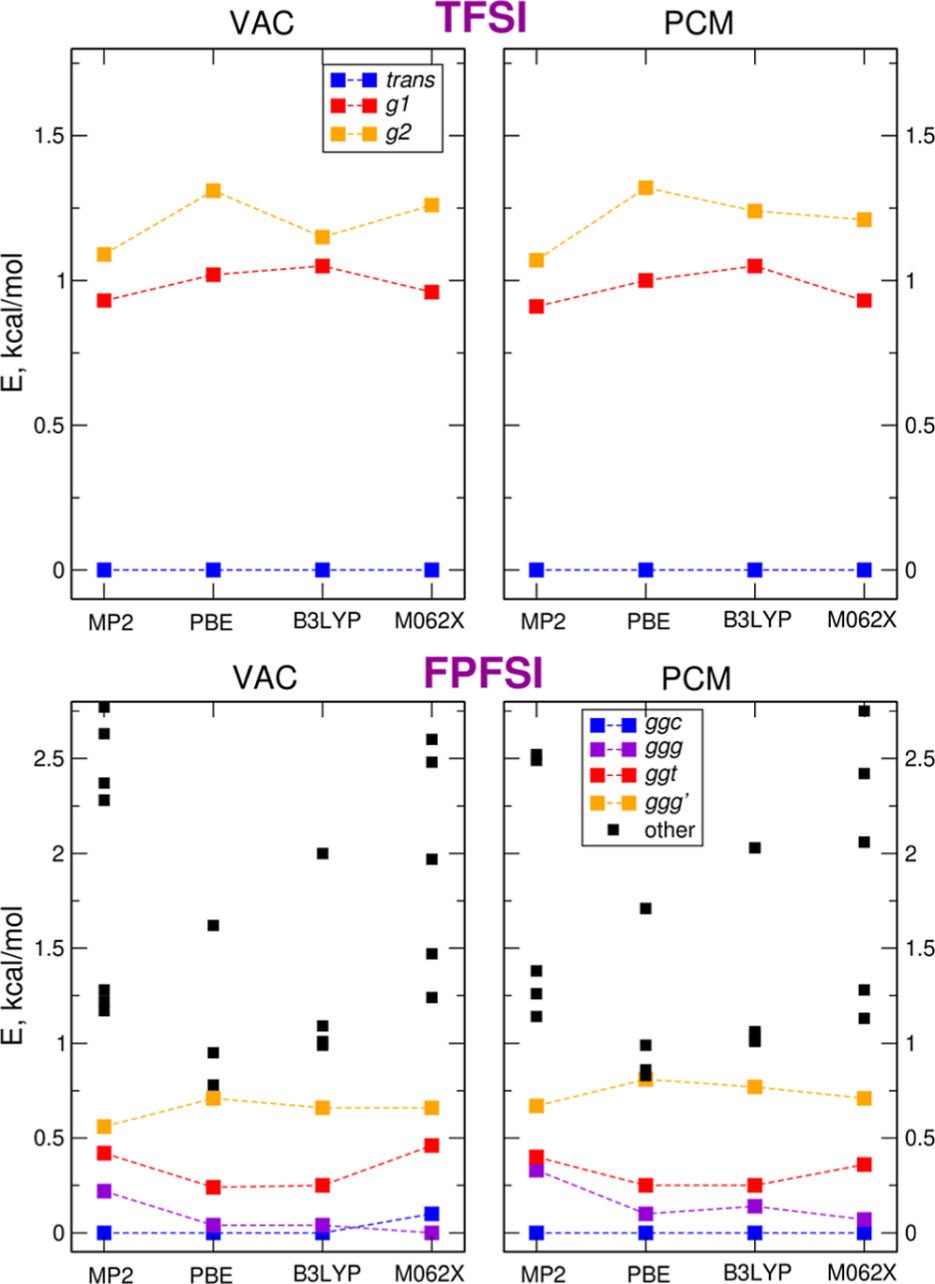
Correlation diagrams for the energies of conformers of TFSI and FPFSI anions calculated using different methods.

Geometries of the conformations shown in Figure 1 and their ordering according to the potential energy remain mostly unchanged in the calculations using the PCM solvent (cf. Tables S2 and S3). For an easy comparison, in Figure 2, we show a correlation diagram for the structures presented in Figure 1. In the case of FPFSI, several higher-lying conformations corresponding to the energy minima are also marked in the plot. The trans conformer of TFSI is always the most stable, regardless of the computational method and accounting for the solvent, and its separation from the gauche conformations remains fairly constant. The variations of the relative energies for the lowest conformers of FPFSI are in a similar range for all methods. The lowest-energy structure is the *ggc* geometry, except for the M062X calculations in vacuum, where it is about 0.1 kcal/mol higher than the *ggg* structure. As readily seen, there are many more possible conformations of FPFSI, with smaller energy differences than for the TFSI isomer. Although we have not calculated the energy barriers between the different minima, an increased number of conformations stems partially form the rotations in the C_2_F_5_ group, which should be relatively easy. Therefore, one may expect an increasing conformational flexibility of the former anion in the electrolyte, in agreement with the general findings of ref (28). With small energy separations between lowest-energy FPFSI conformations we can also conclude that there are no major differences between the methods (MP2 or DFT) tested here.

Partial atomic charges for both anions were obtained from the fit to the electrostatic potential at the MP2/aug-cc-pVDZ level in vacuum (using the MP2 density) according to the Merz–Singh–Kollman scheme with 10 layers of points and 10 points per unit area used in the fitting. Calculated charges are shown in Table S4. Comparing the average values, we may note that the charges on oxygen atoms (the most relevant for the complexation of simple cations) are almost the same: −0.47 and −0.45 e in TFSI and FPFSI, respectively. The most noticeable changes are in the charges on fluorine atoms. The average charge in TFSI is −0.14 e and increases to −0.24 e for the F1 atom in FPFSI. The charge −0.14 e on the F3 atom is similar, and the charge −0.11 e on F2 is slightly smaller compared to F atoms in TFSI. Another quite large difference is observed for C atoms: the charge 0.33 e in TFSI changes in FPFSI to 0.11 e and 0.39 e for C(F2) and C(F3) atoms, respectively. The carbon atoms are hidden in the core of the anion, and it is well-known that the charges of buried atoms obtained from the electrostatic potential are less reliable. However, the charges on the carbon atoms are less important for interactions with other ions. Regarding the electric properties of both anions, we should also mention that the polarizabilities of both ions in MP2 calculations are practically the same. In vacuum, the isotropic polarizabilities are 15.2 and 15.0 Å^3^ for TFSI and FPFSI, respectively; these values increase in the PCM solvent to 18.2 and 17.9 Å^3^.

**Figure 3:**
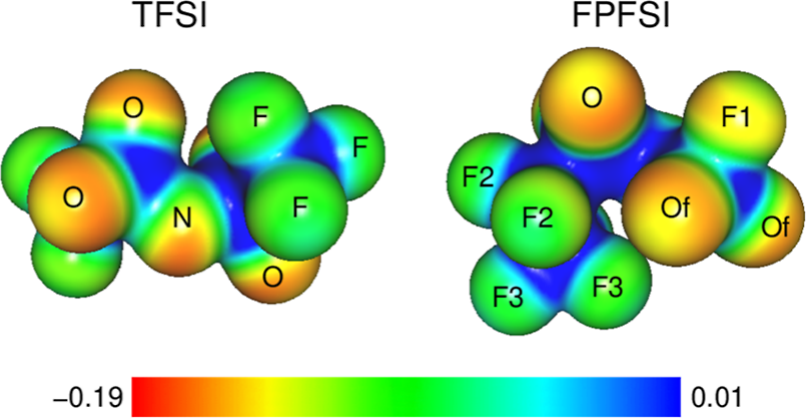
Electrostatic potential (in a.u.) of TFSI and FPFSI anions from MP2/aug-cc-pVDZ calculations, plotted at the 0.02 isodensity surfaces.

In Figure 3, we show the electrostatic potential of trans-TFSI and *ggc*-FPFSI calculated from the MP2 density at the MP2/aug-cc-pVDZ level in vacuum. Indeed, the potential around O atoms in both ions is similar, and the more negative values on the F1 atom of FPFSI can be easily recognized. From these results, we can conclude that both ions will coordinate simple cations, like Li^+^, preferably via oxygen atoms, but there could also be an increased probability of possible Li^+^–F1 interaction in the case of FPFSI anions. We will verify this hypothesis through analysis of calculated cation–anion structures.

**Figure 4:**
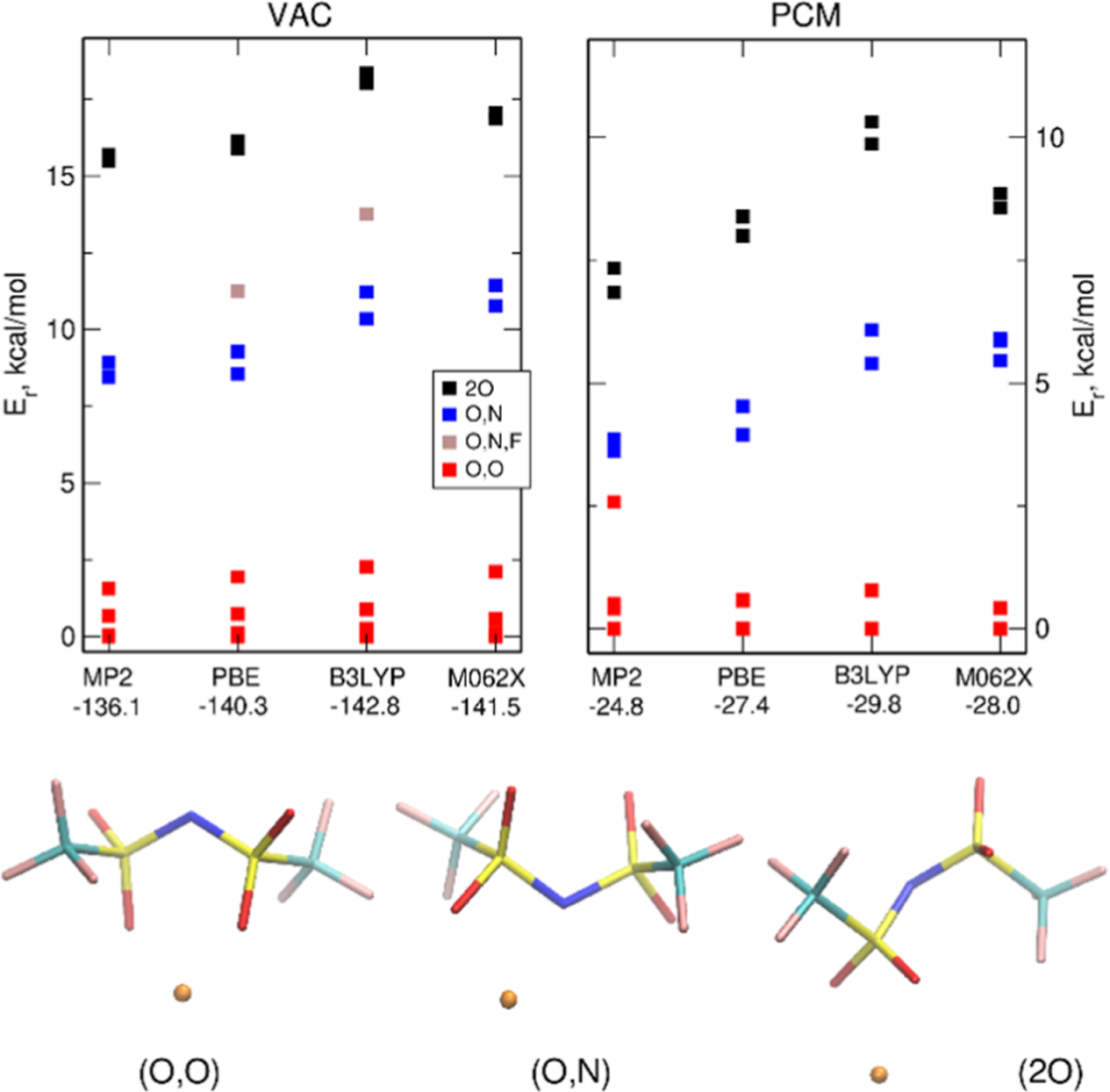
Relative energies of Li–TFSI complexes and the geometries of the most stable structures of each type. The most negative complexation energy, *E*_c_ (in kcal/mol), is indicated for each method.

Relative energies *E*_r_ of different Li–TFSI complexes are shown in Figure 4. The complexation energies *E*_c_ are calculated as
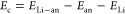
where *E*_x_ stands for the energy of species x (x = Li, an, Li–an), and *E*_an_ is calculated for the most stable conformation of the anion. The most negative complexation energy is indicated for each computational method, and *E*_c_ for other ion pairs can be thus obtained by adding *E*_r_ to this value. Three major types of Li^+^ coordination were found: (O,O)—the most stable bidentate configuration, where the cation interacts with two O atoms from two SO_2_ groups, (2O)—with Li^+^ interacting with two O atoms from the same SO_2_ group, and (O,N)—with Li^+^ coordinated to one O atom and to the N atom. In vacuum calculations using PBE or B3LYP functional, an (O,N,F) type of complex was found, with the cation coordinated to three atoms. For convenience, a summary of parameters for the most stable complex of each type in MP2 calculations is collected in Table 1; the geometries of these complexes in the solvent are shown in Figure 4. In vacuum calculations, (O,N) complexes are about 10 kcal/mol higher than (O,O) structures in agreement with the DFT calculations in ref (15). The (2O) structures are about 15 kcal/mol above the most stable (O,O) complex. In the PCM calculations, the energy differences between the main types of structures are reduced about twice. In the most stable structure of each type, the TFSI anion is in the trans conformation (cf. Table 1), as shown in earlier works.^15^

**Table 1:** Energies, Complexation Energies, and Selected Geometrical Parameters for the Most Stable Li–Anion Complexes of Each Type Calculated at the MP2/aug-cc-pVDZ(PCM) Level

TFSI					
coord. type	*E*_r_, kcal/mol	*E*_c_, kcal/mol	distances, Å	ϕ(CSSC), °	ϕ(CSNS),°
(O,O)	0.0	–24.8	1.983, 1.983^a^	179.4	96.4, 96.5
(O,N)	3.6	–21.2	2.117, 2.276	165.4	86.7, 92.1
(2O)	6.9	–17.9	2.218, 2.340	172.3	89.3, 96.5

FPFSI						
coord. type	*E*_r_, kcal/mol	*E*_c_, kcal/mol	distances, Å	ϕ(FSNS),°	ϕ(SNSC),°	ϕ(NSCC),°
(O,Of)	0.0	–23.3	2.017, 2.024	64.4	–98.3	–37.2
(O,N)	3.1	–20.1	2.143, 2.212	77.5	75.9	141.6
(Of,N)	3.7	–19.6	2.198, 2.188	73.1	97.1	–177.6
(O)	4.9	–18.4	1.973	76.8	95.2	–171.3
(2O)	6.0	–17.3	2.147, 2.502	78.8	82.7	30.5
(2Of)	7.0	–16.3	2.240, 2.407	72.9	96.6	–170.8
(Of)	8.5	–14.8	1.967	92.2	–76.2	–164.6

a Distances from Li^+^ to the coordinating atoms indicated in the coordination type.

**Figure 5:**
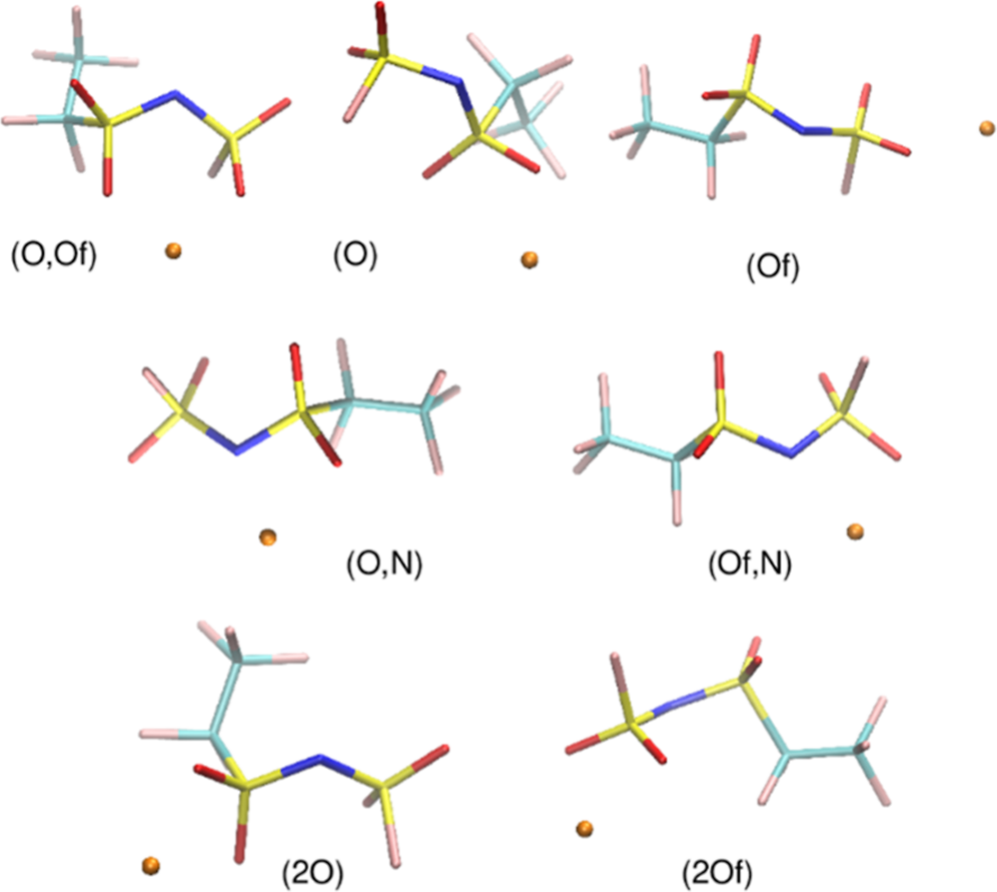
Geometries of the most stable Li–FPFSI structures of each type calculated at the MP2/aug-cc-pVDZ level in the PCM solvent.

**Figure 6:**
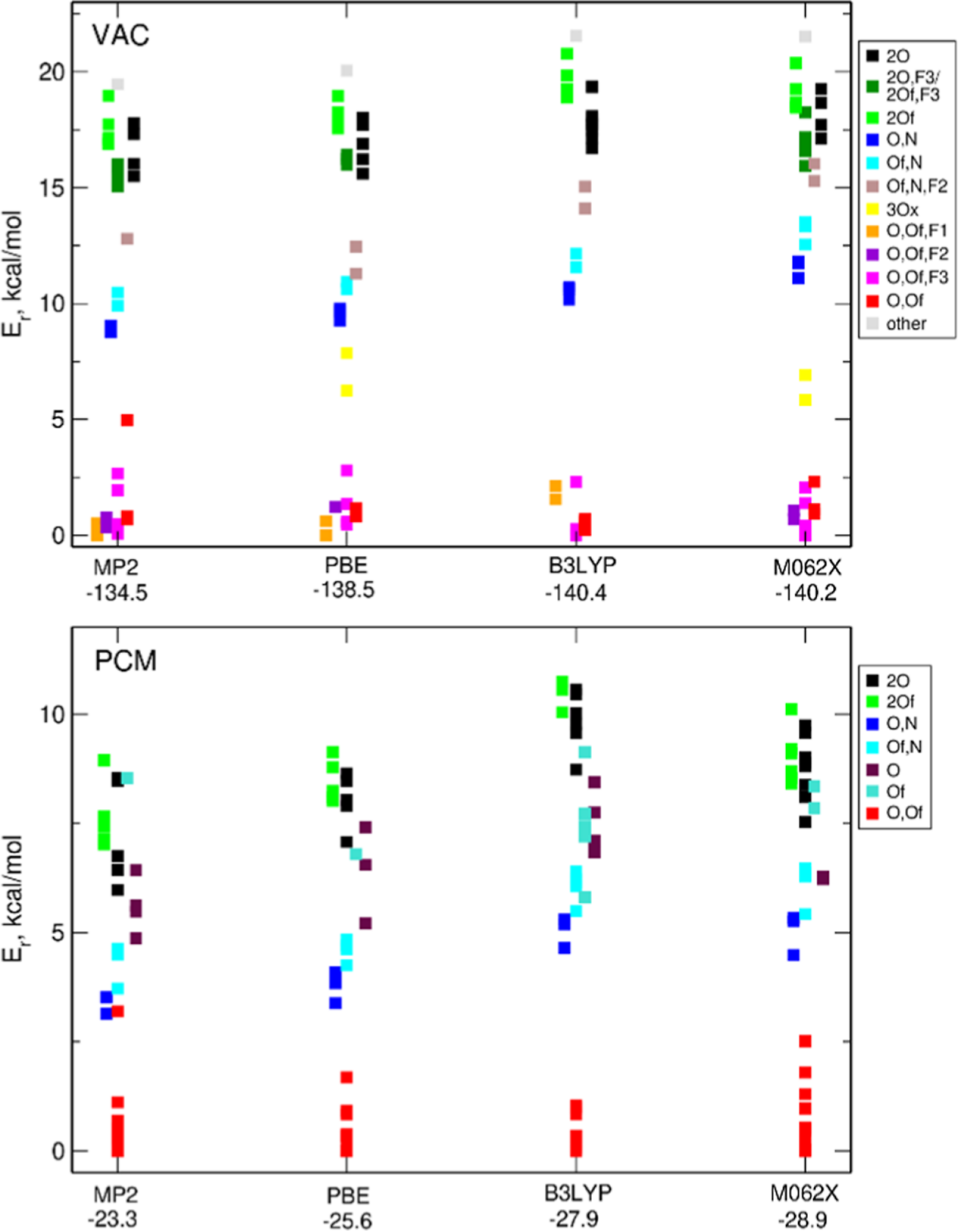
Relative energies of Li–FPFSI complexes and the geometries; the most negative complexation energy *E*_c_ (in kcal/mol) is indicated for each method.

The case of Li–FPFSI pairs is much more complicated because there are two types of oxygen and three types of fluorine atoms. We use the same convention as above to label the structures, indicating the type of atoms to which the cation is coordinated. Geometries of the most stable pairs in the PCM calculations are displayed in Figure 5, the plot of relative complexation energies is shown in Figure 6, and the parameters of complexes are listed in Table 1. In the Supporting Information, we show the geometries of Li–FPFSI pairs obtained in vacuum (Figure S2) and the statistics of Li-anion distances for both anions (Figure S3).

In vacuum calculations, the most stable are the structures with Li^+^ coordinated to two oxygen atoms: (O,Of), which corresponds to the (O,O) geometry of the Li–TFSI pair, and the structures (O,Of,Fx) engaging two oxygen atoms and one fluorine atom (in the following Fx and Ox stand for F or O atom of either type). In MP2 and PBE structures. the fluorine may be any of F1, F2, F3, and the (O,Of,F1) complex has the lowest energy. In M062X calculations the (O,Of,F3) structure is the most stable, in agreement with findings of ref (29), in which this functional was employed. The differences between (O,Of) and (O,Of,Fx) configurations are small (within 1.5 kcal/mol) and depend on the computational method. Similarly to TFSI, there are two more sets of complexes at higher energies: one stemmed from (Ox,N) configurations and the other of (2Ox) parentage—at the highest energies. In both sets, there are also some geometries with a Li–Fx interaction.

This picture changes quite significantly in the solvent, where no structures with Li–Fx coordination were found. Accordingly, the most stable pairs are in the (O,Of) configuration, as found in ref (28). Another type of complexes (Ox), with the cation attached to a sole oxygen atom, is located on the energy scale between (Ox,N) and (2Ox) structures. There are, indeed, some Li–FPFSI complexes with Li–F1 interactions, as predicted from the electrostatic potential, and few structures with cation coordinated to F2 or F3 atom (owing to the flexible FPFSI anion). Nevertheless, all these structures were found only in vacuum. In the solvent, both anions exhibit the same coordination modes, and only the number of possible distinct Li–FPFSI structures increases due to the inequivalence of atoms in an asymmetric anion. In vacuum and in PCM calculations, energy differences between the (O,Ox), (Ox,N), and (2Ox) types are similar for TFSI and FPFSI.

The binding energies of Li–FPFSI pairs are only slightly lower than those for Li–TFSI complexes. At the MP2 level in vacuum, *E*_c_ is −136.1 and −134.5 kcal/mol for TFSI and FPFSI anion, respectively. These values are reduced in the solvent to −24.8 and −23.3 kcal/mol. A similar trend is observed in the DFT results. Therefore, the general conclusion of ref (28) about weaker Li^+^ binding to asymmetric anions is confirmed by the detailed analysis, but the actual difference calculated for TFSI/FPFSI isomeric anions is very small.

While the implicit solvent used in calculations for cation–anion complexes can account fairly well for the electrostatic screening, affecting the interaction energies, some effects are missing in the model. In particular there is no explicit Li^+^–solvent complexation and no charge transfer between the solvation shell and the cations, known to be important in electrolyte solutions.^18,49^ These features can be modeled when larger systems (containing explicit solvent molecules) are investigated, preferably using ab initio MD simulations.

Regarding the comparison of MP2 and DFT, one can observe that all DFT functionals tested here predict stronger Li–anion interactions. The PBE is the closest to MP2, and hybrid functionals yielded the largest stabilization of the ion pair. It can also be noted that the energy separation between the (O,Ox) and (Ox,N) structures increases in DFT, and it is the largest in the B3LYP calculations. Accordingly, the Li-Ox or Li–N distances in PCM calculations (Figure S3) are the largest in the MP2 results and decrease when the DFT methodology is used. The Li-Ox distances are similar in all three functionals used, whereas the Li–N distances obtained in the M062X calculations are larger than those for PBE or B3LYP and are quite close to the MP2 results. However, the probability of the Li–N coordination in an electrolyte will be marginal because of the much higher energy of complexes. The overall agreement of structural data and relative energies of complexes between MP2 and DFT is the best for the PBE functional. Therefore, using the relatively computationally cheap GGA PBE functional instead of more expensive hybrid functionals in ab initio MD simulations seems a rational choice.

### 3.2 Vibrational Spectra

Vibrational spectroscopy is used to monitor the conformations of molecules and ions in solutions and to detect the ion–ion interactions in electrolytes; therefore, comparing the spectra of both ions can provide some suggestions for the analysis of experimental data. The vibrational spectra (IR and Raman) in harmonic approximation were calculated for selected lowest-energy conformations of TFSI and FPFSI anions. The results obtained in vacuum and in the PCM solvent are compared in Figures S4 and S5 in the Supporting Information. There are differences observed in the spectra of different conformers, nevertheless, we will not discuss this issue here, focusing instead on the effect of the computational method and the shifts induced by interactions. An analysis of the conformational effects in the spectra of TFSI has been presented, e.g. in refs (10,12).

The spectra of both anions are similar, although the spectra of FPFSI are richer in features due to different structure and lower symmetry of the anion. For both anions, the spectra calculated in PBE and B3LYP functionals are shifted to lower wavenumbers compared to the MP2 results. The shift is larger for PBE, amounting to about 100 cm^–1^. On the other hand, the M062X spectra are about 30 cm^–1^ blue-shifted with respect to MP2. Accounting for the solvent in the PCM calculations results in a small red-shift of the spectrum and an increase in intensity. The measured frequencies of TFSI vibrations in PEO solvent^9^ are higher than the results of our calculations; therefore, the best agreement to the experiment is obtained in M062X DFT calculations and then in the MP2 method. The PBE functional yielded the most underestimated band positions. We can expect that the same relation holds for the FPFSI spectra. Although the poor performance of PBE in reproducing experimental frequencies may seem problematic in the AIMD simulations employing this functional, this is not a significant limitation. In studies of ion complexation effects, frequency shifts are more important than the actual band positions, and the former are quite well described in PBE calculations, as we will see later.

**Figure 7:**
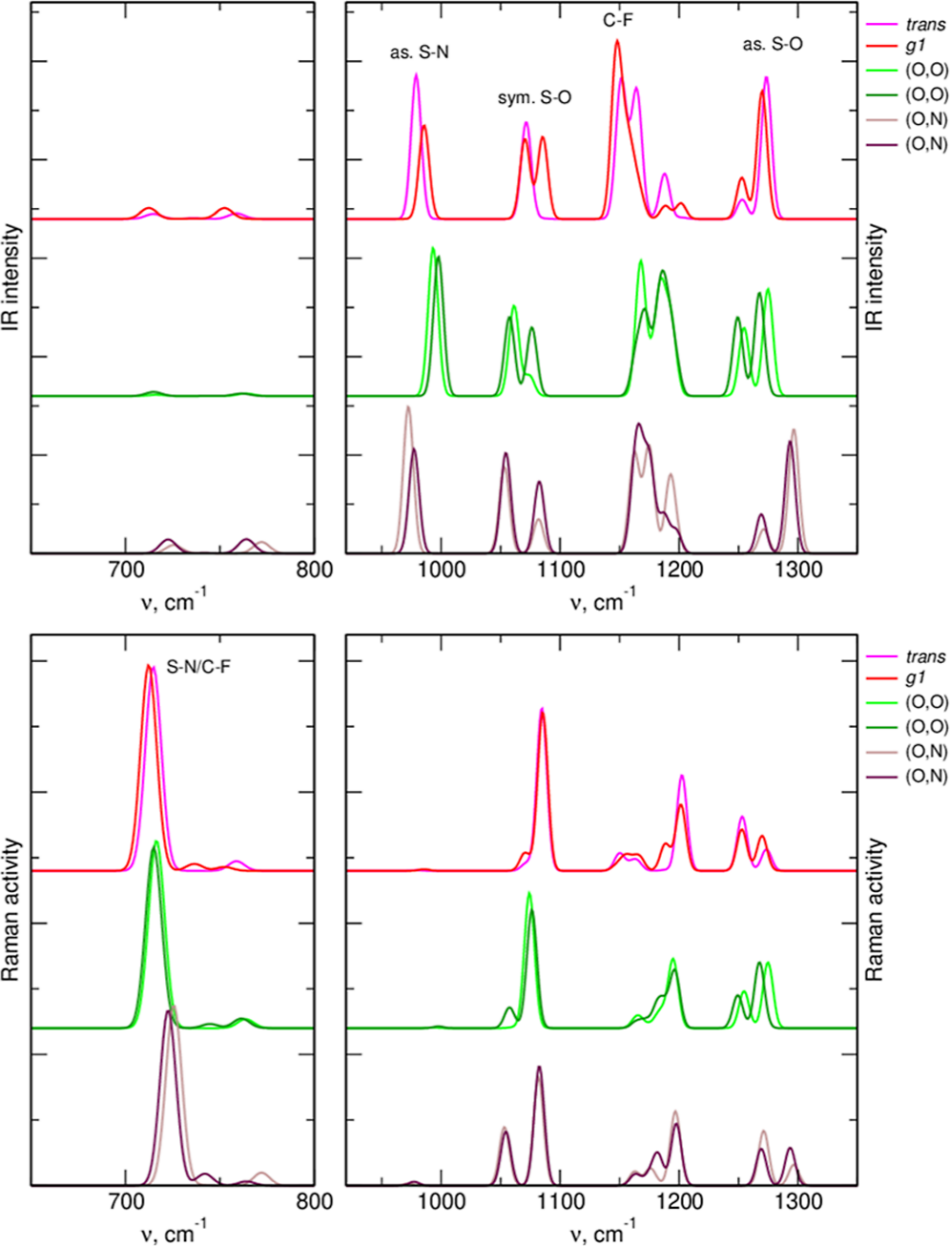
Vibrational spectra of lowest-energy conformers of free TFSI anion and the most stable Li–TFSI complexes with different types of coordination.

**Figure 8:**
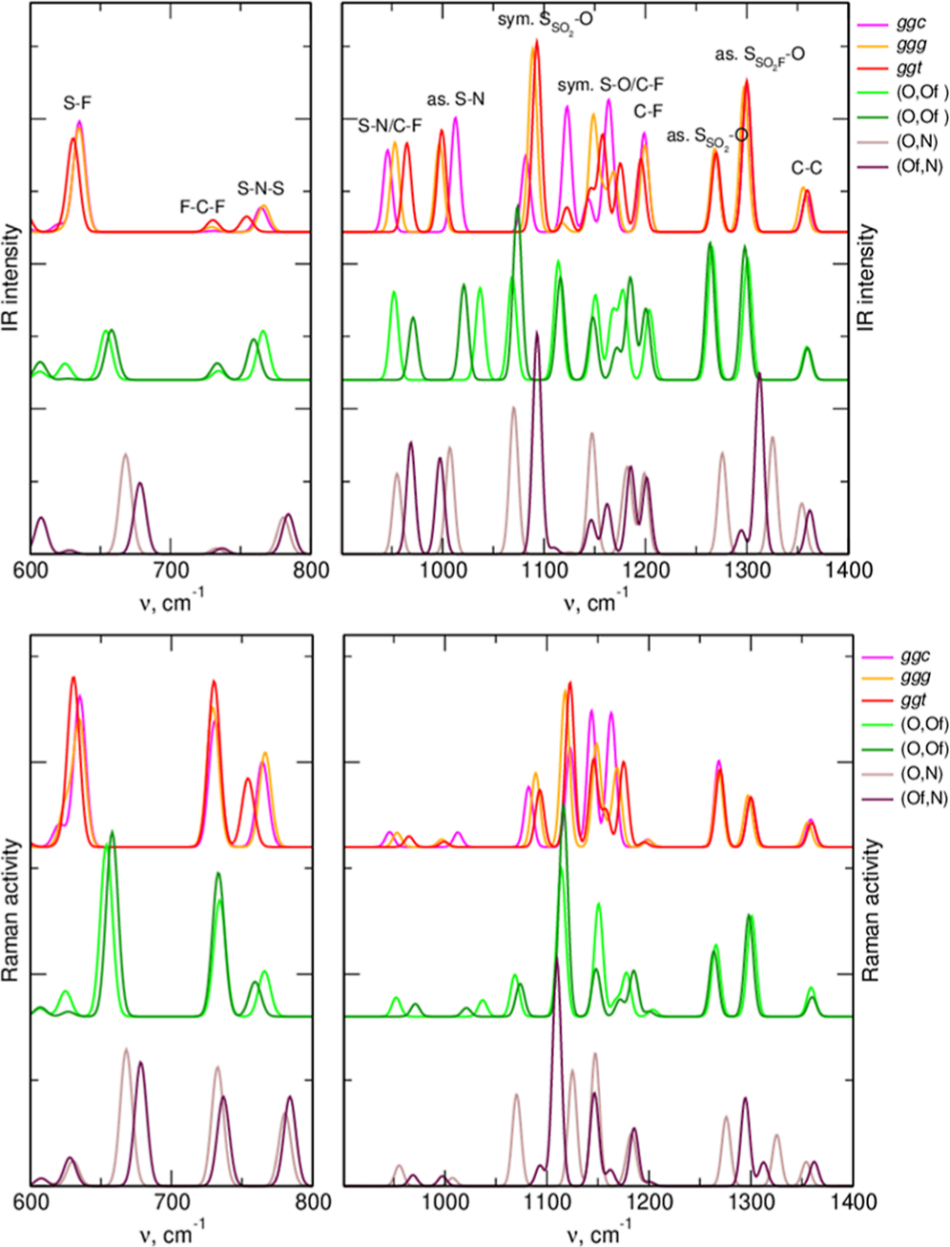
Vibrational spectra of lowest-energy conformers of free FPFSI anion and the most stable Li–FPFSI complexes with different types of coordination.

In Figures 7 and 8, we compare the MP2(PCM) spectra obtained for free anions and two lowest-energy ion pairs with (O,Ox) and (O,N) interactions. The TFSI anion coordinates the Li^+^ cation via the oxygen atoms, and the changes in the S=O stretching frequencies are therefore expected upon complexation. As seen in Figure 7, bands corresponding to both symmetric and asymmetric O=S=O modes in (O,O) complexes are shifted to lower wavenumbers. Another band sensitive to interactions is the Raman active vibration at about 750 cm^–1^, with the frequency increasing upon Li^+^ coordination. Both these spectral regions are used to monitor the anion–Li^+^ interactions in the experiments.^14,16^

According to Figure 8, S=O stretches and S–F vibrations in the range 600–700 cm^–1^ can also be used to detect the Li^+^ coordination in the FPFSI-based electrolytes. In particular, the latter band is advantageous because (unlike TFSI) it is IR and Raman active, and the complexation-induced shift is larger than that calculated for TFSI. In both anions, also the asymmetric S–N stretching vibration at about 1000 cm^–1^ responds with a blue-shift to the (O,Ox) coordination of the cation. It can also be noted, that the effects of the (Ox,N)-type coordination depend on the vibration. For symmetric O=S=O vibrations and the C–F/S–F band between 600 and 750 cm^–1^, the shift is in the same direction as for the (O,Ox) coordination. On the other hand, for asymmetric S=O and S–N vibrations, the changes caused by both types of coordination are opposite. However, as the probability of formation of the (Ox,N) complexes in the real electrolyte is negligible, these observations are of little practical importance.

### 3.3 Structure of Electrolytes from AIMD Simulations

**Figure 9:**
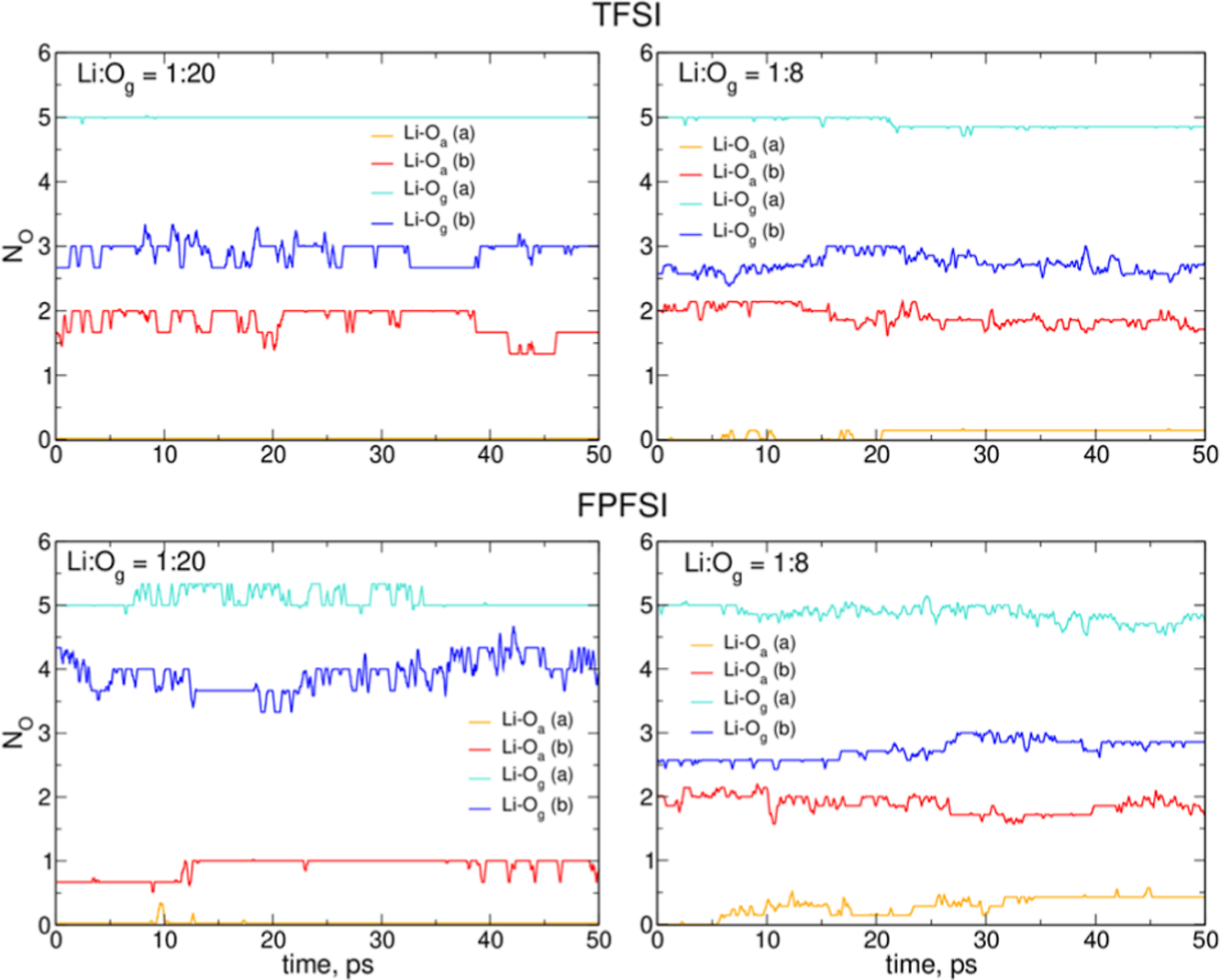
Changes in the average number of O_a_ and O_g_ atoms coordinating the Li^+^ ions in the AIMD simulations of electrolytes.

To assess the evolution of the structure of the Li^+^ coordination shell during the AIMD simulations of LiTFSI(LiFPFSI)/G4 electrolytes, we plotted in Figure 9 changes in the average number of O_a_ and O_g_ atoms interacting with the cation. Here, O_a_ and O_g_ denote the oxygen atom from an anion or a tetraglyme molecule, respectively, and the interaction was counted when the Li–O distance was less than 3 Å.

In the systems with Li cations initially coordinated to G4 molecules (type *a* structures), at the beginning of the trajectory, Li^+^ ions interacted solely with 5 O_g_ atoms. Coordination changes in this type of electrolytes are very slow, and after 50 ps of simulations the average values changed by no more than 0.4 (the largest change in the 1:8 LiFPFSI system). Although the average coordination numbers for structures *a* and *b* tend to converge, which is better pronounced at the 1:8 concentration, the convergence is very slow due to the viscosity of the salt solutions.

**Figure 10:**
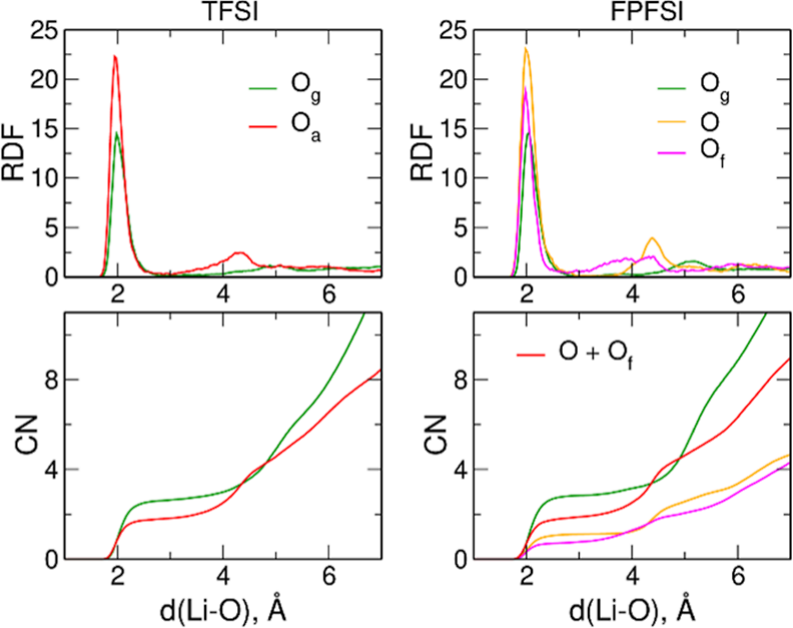
Radial distribution functions and integrated RDFs in the 1:8 electrolytes of type b.

Radial distribution functions (RDFs) and integrated RDFs for Li–O atom pairs in the 1:8 electrolytes of type *b* are shown in Figure 10. The first maximum in the Li–O_g_ RDF appears at 2.00 or 2.05 Å for the system with TFSI or FPFSI anions, respectively. The maximum corresponding to Li–O_a_ pairs is located at slightly lower distances −1.95 Å for TFSI anions or 1.98–1.99 Å for O atoms from SO_2_ and SO_2_F groups of FPFSI anions. The larger distances observed for Li–O_FPFSI_ RDFs indicate that Li^+^ interactions with FPFSI are somewhat weaker than binding to TFSI.

The integrated RDFs show that Li cations interact mainly with tetraglyme molecules regardless of the anion. The Li^+^ coordination numbers (CNs) in the LiTFSI solution, obtained as the number of O atoms within 3 Å from the cation, are 2.63 and 1.83 for O_g_ and O_a_ atoms, respectively. In the LiFPFSI-based electrolyte, the CN for O_g_ atoms increases to 2.84, with simultaneous small increase of the total CN to O_a_ atoms to 1.88, confirming the conclusion drawn from Figure 9, that in the solutions with FPFSI salt, the degree of Li^+^ coordination to glyme molecules is increased. It can also be noted that the CNs to O and Of atoms of FPFSI anions are similar. Although this suggests the (O,Of) type of coordination, we will see later that this is only partially true.

Low Li^+^ coordination to TFSI anions in glyme solutions has already been documented in the literature from classical or ab initio MD simulations.^17−20^ The difference between the Li CNs to anions and to glyme oxygens is particularly well pronounced for G4 solutions,^17,19^ because the G4 molecule wraps around the cation, effectively forming the solvates with CN = 5. The average number of O_g_ atoms coordinating the cation found in our simulations is smaller than in the classical MD simulations for LiTFSI/G4 solutions;^20^ accordingly, our data show larger numbers of coordinating O_TFSI_ atoms. To some extent, the difference may be due to a different methodology (AIMD vs force field-based MD), but the most obvious cause is that the time of AIMD simulations is too short to allow the complete equilibration of the system. Therefore the CNs are different from the “true” values in the fully relaxed systems. On the other hand, positions of the RDF maxima are more reliable as even the short trajectories are sufficient to adjust the distances in the first solvation shell.

In addition to Li–O coordination, we also examined the Li–F RDFs, shown in Figure S6 in the Supporting Information. Regardless of the anion, the RDF values in the range 2–3 Å are very small, and with the largest Li–F CN calculated at 3 Å less than 0.07 (for the 1:8 LiFPFSI electrolyte), we can conclude that there are no appreciable direct Li–F interactions in the studied electrolytes. The peaks at 4 Å and above correspond to Li–F distances in configurations in which the Li^+^ ion is coordinated to one or two Of atoms. In particular, the maximum in the Li–F1 RDF obtained for the 1:8 LiFPFSI electrolyte arises from the structure of this system (shown in Figure S7 in the Supporting Information), where one of the Li^+^ ions interacts with two Of atoms from two different FPFSI anions and one O atom from the third anion forming a [Li(FPFSI)_3_]^2–^ quadruplet. However, with small number of ions in the system is hard to assess how often such a configuration can appear in the entire population of ions. Lack of direct Li–F coordination in the AIMD data for LiFPFSI electrolytes is consistent with the QC data in Section 3.1, showing that such structures are not stable in the solvent.

To complete our analysis of Li coordination, we calculated the probability of different local Li environments, that is, combinations of different numbers of interacting O_g_ and O_a_ atoms (O atoms within the 3 Å distance from the cation). The data are shown in Figure S8 in the Supporting Information. Because of the uncertainty of the results for the 1:20 systems, easily affected by the configuration of a single ion in the system with just three ion pairs, we note only that in the 1:20 LiFPFSI electrolyte, about half of Li^+^ ions are not coordinated to any anion and interact solely with 4–6 G4 oxygen atoms. In the LiTFSI electrolyte no such cations are found. At the 1:8 ratio, the most probable is the coordination to one O_a_ atom and three O_g_ atoms in the LiTFSI solution (28%) or to one O_a_ atom and four O_g_ atoms in the LiFPFSI electrolyte (39%). The overall probability distribution in the LiFPFSI electrolyte is shifted toward a lower number of O_a_ atoms and a higher number of coordinating O_g_ oxygens. In the LiTFSI electrolyte, there are Li^+^ ions (26%) interacting with 3 O_a_ atoms from two different ions; similarly, the configuration in which the two coordinating O_a_ atoms come from two anions (15%) is more probable than the bidentate coordination to one anion (10%), and 47% of Li^+^ is engaged in monodentate coordination. In the LiFPSI solution, two coordinating O_a_ oxygens are almost always from one anion (28%). Nevertheless, there is also a possibility of coordination to three O_a_ atoms from two or three anions: 15% and 10%, respectively; these geometries correspond to the coordination shell of one of the Li^+^ cations, shown in Figure S7. The monodentate coordination to a single anion is the most probable (42%). Similar Li^+^ CNs for the O and Of atoms (Figure 10) resulted therefore mainly from approximately equal probabilities of (O) and (Of) configurations. In the bidentate coordination in the FPFSI electrolyte, the average number of O_g_ oxygens is increased compared to the LiTFSI solutions. This results probably from the asymmetric structure of the FPFSI anion, allowing for more easy access to the G4 molecule when the cation is coordinated in a (O,Of)-type structure. These data on Li coordination shells indicate that in the LiFPFSI electrolytes, the degree of interactions with anions is reduced in favor of Li–G4 interactions, in agreement with QC results showing that Li^+^ binding to FPFSI is slightly weaker than interactions with TFSI. Finally, we should mention that the probability of cation interactions with TFSI in our AIMD simulations is larger than in the classical MD results,^20^ in accord with the differences in the average CNs.

### 3.4 IR Spectra from AIMD Simulations

**Figure 11:**
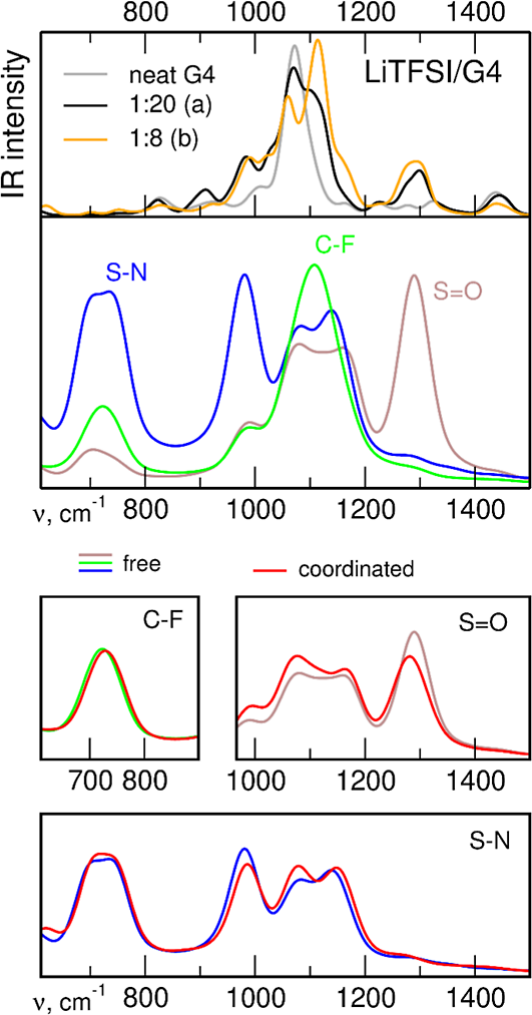
Simulated IR spectra of LiTFSI/G4 electrolytes and neat G4 (top panel); power spectra of selected TFSI oscillations (middle panel); changes in the power spectra upon Li coordination (bottom panels).

**Figure 12:**
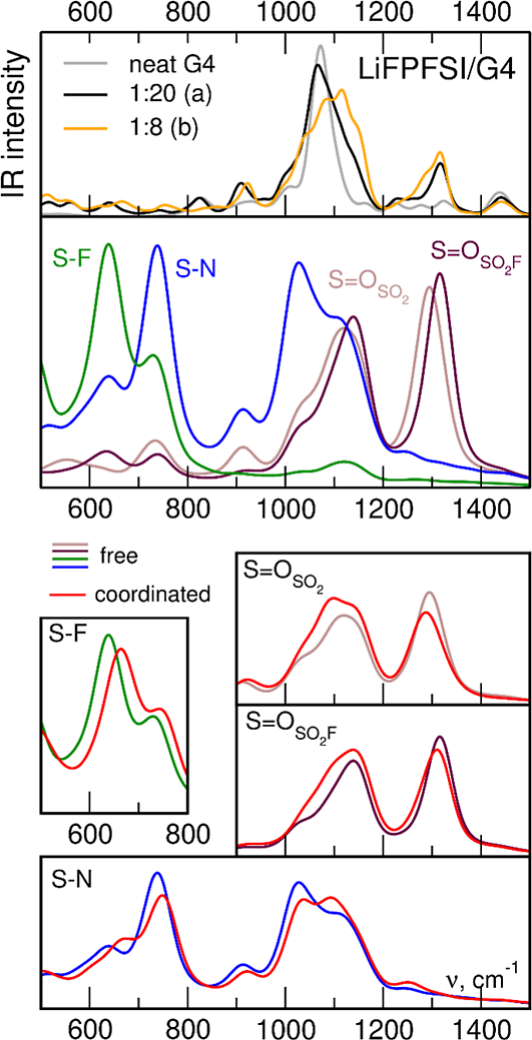
Simulated IR spectra of LiFPFSI/G4 electrolytes and neat G4 (top panel); power spectra of selected FPFSI oscillations (middle panel); changes in the power spectra upon Li coordination (bottom panels).

To assess the effects of anion–cation interactions in the IR spectra, we compared the spectra for the two systems with the largest difference in coordination: the 1:20 electrolyte of type *a*, where almost all anions are free, and the 1:8 electrolyte of type *b*, in which most anions interact with Li^+^ cations. Band assignments in the AIMD-based spectra and the analysis of coordination-induced frequency shifts are facilitated by the power spectra of bond length oscillations, providing information about the contributions of local modes to the total spectrum. We used such an analysis, e.g. to correlate ion–solvent interactions with IR spectra of salt solutions in cyclic carbonates.^50^^,^^51^ In the experimental spectra, interactions manifest quite often as changes in the width or in the shape of the band, and the effects of ion coordination are visible only after the spectrum is decomposed into individual bands corresponding to free and interacting molecules/ions. Analysis of the power spectra helps to determine the frequencies at which these bands are expected to appear. For the assignments of anion bands in the spectra of the electrolytes studied in this work, we used the FTs of bond lengths averaged over all free anions in the 1:8 *a* electrolytes. To detect the shifts caused by interactions, we compared these data with power spectra averaged over coordinated anions in the systems of type *b* at 1:8 concentration. The calculated IR and power spectra for LiTFSI/G4 and LiFPFSI/G4 electrolytes are shown in Figures 11 and 12, respectively; the spectrum of neat G4 is shown for comparison. Additionally, power spectra from the velocity autocorrelation function are presented in Figures S9 and S10 in the Supporting Information.

The three most intense bands in the IR spectrum of the 1:20 LiTFSI type *a* electrolyte appear at 984, 1070, and 1299 cm^–1^. The band at 1444 cm^–1^ corresponds to G4 vibrations. Based on the FTs of bond lengths, the band at ∼990 cm^–1^ can be attributed to S–N stretching vibrations, the 1299 cm^–1^ band is due to S=O oscillations, and in the region of the middle band, the S=O, C–F, and S–N vibrations of the anion are mixed, overlapping the most intense band of G4. This assignment agrees with the QC data in Figure 7; the upper S=O band is due to asymmetric stretches, whereas the lower corresponds to symmetric vibrations. Power spectra in Figure S9 lead to similar conclusions about the frequency ranges in which the contributions from the anion and the solvent appear in the spectrum.

In the spectrum obtained at higher LiTFSI concentration, the S–N band upshifts to 991 cm^–1^; the maximum of the asymmetric S=O band is shifted to 1294 cm^–1^, with accompanying intensity shift toward the low energy side of the band. The main maximum of the symmetric S=O vibrations appears at 1114 cm^–1^, and an additional peak develops at 1060 cm^–1^. These observations agree with the general expectations from MP2 results for an isolated ion pair (Figure 7), predicting a blue-shift of the S–N band and red-shifts of the S=O vibrations; however, the changes of S=O frequencies in AIMD-based spectra are smaller.

Using FTs of bond lengths, we can relate these changes in the IR spectra to the local coordination patterns of TFSI anions; the red curves in the lower panels of Figure 11 show the power spectra averaged over all S–N and C–F bonds in the anions interacting with cations and over those S=O bonds which are Li^+^ coordinated. We should remember that these plots show only contributions of frequencies to the vibrational modes but not the IR intensity. The blue-shift of the S–N maximum and the red-shift of the asymmetric S=O vibrations (both by 6–7 cm^–1^) are well noticeable.

The case of the band at ∼1100 cm^–1^, corresponding to symmetric S=O stretches (coupled to C–F and S–N oscillations), is more complicated. As seen in Figure 11, the corresponding band in the S=O power spectrum is wide and has some structure. In MP2 calculations (Figure 7), two separate maxima for this vibration are assigned to in-phase and out-of-phase oscillations.^14^ The distribution of the IR intensity (and the power spectra) in this frequency range depend on the conformation of the anion. Therefore, the wide structure of the power spectrum around 1100 cm^–1^ can be attributed to the TFSI anions in different conformations and/or changing the conformation during the AIMD simulation. In Figure 11, the S=O power spectrum at 1100 cm^–1^ shows some redistribution of frequencies for S=O bonds coordinated to cations, but the overall effect is rather very limited. These results agree with the findings of ref (14), where it was concluded that the sensitivity of S=O oscillations in the vibrational spectrum of TFSI to the interactions with Li^+^ is unexpectedly small, as per the bond directly involved in the coordination of the cation.

A Raman-active vibration at 740 cm^–1^ is often used in spectroscopic studies of TFSI interactions.^14,16^ The MP2 spectra in Figure 7 indicate a clear blue-shift of this band upon Li^+^ coordination. In our AIMD-simulated IR spectrum in Figure 11, there is, of course, no corresponding band, but the power spectra can be used to analyze the frequency changes. Indeed, both S–N and C–F power spectra indicate a vibration at 720–735 cm^–1^. The power spectra for coordinated anions are blue-shifted by 5–10 cm^–1^ with respect to the maxima obtained for free TFSI. Although the shift is not very large, it is readily visible in the spectrum, confirming that the 740 cm^–1^ Raman band can be used to monitor the coordination status of TFSI ions in solution. Its value agrees well with the coordination-induced upshifts of 6 or 5 cm^–1^ observed for the 740 cm^–1^ Raman band of TFSI in a Li salt solution in an ionic liquid^14^ or in LiTFSI/G4 electrolyte.^16^ However, we should note that a recent study indicates that the picture of only two (uncoordinated and coordinated) bands is an oversimplification and the electrolyte spectrum should be deconvoluted into more bands, corresponding to different degree of Li–TFSI interaction/aggregation.^52^

Three bands from the IR spectrum of the 1:20 LiFPFSI type *a* electrolyte (Figure 12) are of our interest: a weak maximum at 640 cm^–1^, the most intense peak at 1066 cm^–1^, and the band with a maximum at 1317 cm^–1^. The power spectra in Figure S10 and the FTs of bond lengths allow us to assign these features to the FPFSI vibrations. The two upper bands correspond to S=O stretches. In the 1:8 type *b* electrolyte, with more FPFSI–cation interactions, these bands behave similarly to the peaks in the LiTFSI electrolytes. The maximum of the highest peak is unaffected, but the IR intensity increases in the low-energy shoulder of the band. Similarly, for the most intense band, the main maximum shifts to higher energies, but a small shoulder develops below, at about 1050 cm^–1^. We can attribute these red-shifted features to the S=O bonds in the FPFSI anions interacting with Li^+^.

Power spectra calculated for S=O oscillations confirm the above conclusion. In both bands, corresponding to symmetric and asymmetric S=O stretches, oscillations of S=O bonds in the SO_2_F group are at frequencies higher than the S=O oscillations in the SO_2_ group, in agreement with MP2 data in Figure 8. Power spectra calculated for S=O bonds involved in Li^+^ coordination are shifted to lower wavenumbers. The shifts for the SO_2_ group are similar in the symmetric and asymmetric vibrations (−20 and −10 cm^–1^, respectively), whereas the maximum of the symmetric mode of SO_2_F is not shifted. For the asymmetric SO_2_F mode, shift of the maximum is −7 cm^–1^, which is close to that observed for the SO_2_ group. In general, red-shifts of the S=O vibrations in the FPFSI electrolyte are better pronounced than in TFSI solutions, suggesting that S=O bands can be used to monitor the coordination of the former anions.

Power spectra indicate that the band at 640 cm^–1^ is related to S–F vibrations, as already concluded for the MP2-based spectra in Figure 8. An upshift of the IR intensity is very well pronounced in the spectrum of 1:8 type *b* electrolyte, with the maximum appearing at 666 cm^–1^. A similar blue-shift of 26 cm^–1^ is observed for the power spectrum of S–F vibrations in coordinated FPFSI anions. We can, therefore, confirm the conclusions drawn from QC calculations in Section 3.2, that the S–F band above 600 cm^–1^, IR and Raman active, and exhibiting a clear shift upon cation coordination, seems to be very convenient for experimental studies of FPFSI interactions with lithium cations.

Finally, we can note that the band corresponding to S–N–S deformation, located at 742 cm^–1^ in the IR spectrum of 1:20 LiFPFSI type *a* electrolyte, is another promising candidate for coordination studies of FPFSI. Both the IR spectra and the power spectrum of S–N oscillations show a clear coordination-induced blue-shift of this mode. Although its IR intensity is weak, the band is Raman active (cf. Figure 8); therefore, it can be studied using Raman spectroscopy.

## 4 Conclusions

We computationally compared two isomeric anions, TFSI and FPFSI, using quantum chemical calculations to assess the conformational properties, binding energies, and structure of ion pairs, and performing AIMD simulations to investigate the structure of salt solutions in tetraglyme and their IR spectra.

Asymmetry of the FPFSI anion and the flexibility of C_2_F_5_ group lead to an increased number of low-energy conformations and possible structures of anion–Li^+^ pair. Nevertheless, the preferred structure of an ion pair for both anions is a bidentate coordination of the Li^+^ ion by two oxygen atoms from two SO_2_ groups. Binding of lithium cation to FPFSI anions is weaker than to TFSI, but the difference is relatively small (1.5 kcal/mol in the solvent). These findings agree with an earlier QC study on ion pairs with asymmetric fluorinated anions.^28^

In the G4-based electrolytes with LiTFSI and LiFPFSI salts, the preferred Li^+^ coordination is to oxygen atoms from the solvent molecules. The average number of coordinating O_g_ atoms increases in the electrolytes based on LiFPFSI; therefore, according to our AIMD results, the asymmetric anion reduces ion pairing and promotes cation interactions with ether molecules, in agreement with slightly lower stability of Li–FPFSI ion pairs obtained from QC calculations. However, the AIMD simulations are limited to small systems and short simulation time; therefore, statistics of coordination are poor, and the long-time dynamics is inaccessible, rendering impossible to analyze the effects of rather subtle differences between the two anions in the first-principles MD. To obtain more reliable statistics and some estimates of transport properties (diffusion coefficients, conductivity), classical MD simulations with parametrization tailored to TFSI/FPFSI anions will be necessary. We plan such investigations, based on the QC data obtained in this work, for future research.

According to the analysis of the IR spectra obtained from AIMD simulations, supported by QC calculations for isolated ion pairs, we can suggest that the IR and Raman active S–F stretching vibration of the FPFSI anion in the range 600–700 cm^–1^ exhibits sensitivity to interactions with Li^+^. The band is well separated from other features in the spectrum, and the blue-shift induced by interactions is quite large, making this band a perfect candidate for spectroscopic monitoring the coordination of FPFSI anions in solutions.

## Supporting Information Available

Compositions of simulated systems, energies and structural data for TFSI and FPFSI conformers, partial atomic charges, geometries of LiFPFSI structures in vacuum, Li-anion distances, vibrational spectra of TFSI and FPFSI obtained with MP2 and DFT methods, Li–F RDFs, a sample snapshot of the 1:8 LiFPFSI-b electrolyte, probabilities of different coordination environments, power spectra of LiTFSI/G4 and LiFPFSI electrolytes, Cartesian coordinates of gauche TFSI conformers (PDF)
