# Supplementary material for: Comparative Study of Isomeric TFSI and FPFSI Anions in Li-Ion Electrolytes Using Quantum Chemistry and Ab Initio Molecular Dynamics
Source: J Phys Chem B. 2025 Feb 19;129(9):2560–72. doi: 10.1021/acs.jpcb.4c08414 (PMC11891891; doi:10.1021/acs.jpcb.4c08414)
Supplement: Supplementary file 1 — jp4c08414_si_001.pdf [file jp4c08414_si_001.pdf]

Comparative Study of Isomeric TFSI and FPFSI Anions in Li-Ion Electrolytes Using Quantum Chemistry and Ab Initio Molecular Dynamics

Piotr Kubisiak,[a] Domantas Narkevičius,[b,a] Chiara Nicotri,[a,c] Andrzej Eilmes[a,*]

[a] *Faculty of Chemistry, Jagiellonian University, Gronostajowa 2, 30-387 Kraków, Poland*

[b] *Faculty of Physics, Vilnius University, 3 Universiteto St., LT-01513 Vilnius, Lithuania*

[c] *Department of Applied Science and Technology, Politecnico di Torino, Corso Duca degli Abruzzi 24, 10129 Torino, Italy*

Supporting Information

Table S1. Compositions of Simulated LiTFSI/LiFPFSI Solutions in Tetraglyme (G4).

Li:O$_g$	No. of G4 mols.	No. of ion pairs	Density (g/cm^3)[a]
1:20	12	3	1.160
1:8	11	7	1.316

[a] Experimental densities from Yoshida, K.; Tsuchiya, M.; Tachikawa, N.; Dokko, K.; Watanabe, M. *J. Phys. Chem. C* **2011**, *115*, 18384.

Table S2. Relative Energies and the Structural Data for TFSI Conformers Calculated at the MP2/aug-cc-pVDZ Level.

	vacuum				PCM			
structure	E, (kcal/mol)	ϕ(CSSC), °	ϕ(CSNS), °	# i.f.[a]	E, (kcal/mol)	ϕ(CSSC), °	ϕ(CSNS), °	# i.f.
trans	0.00	169.7	91.2,91.2	0	0.00	170.4	91.7,91.7	0
g1	0.93	39.9	-82.8,123.5	0	0.91	38.4	-82.5,121.7	0
g2	1.09	76.7	-97.4,168.5	0	1.07	75.9	-98.9,168.9	0
cis-1	2.62	0.0	107.4,107.4	1	2.58	0.0	107.2,107.2	1
g3	3.08	-88.3	142.5,142.5	1	3.05	-79.2	147.1,147.1	1
cis-2	4.37	0.0	180.0,180.0	1	3.88	0.0	180.0,180.0	1
trans-2	9.31	180.0	0.0,180.0	2	9.29	180.0	0.0,180.0	2

[a] the number of imaginary frequencies

Table S3. Relative Energies and the Structural Data for FPFSI Conformers Calculated at the MP2/aug-cc-pVDZ Level.

	vacuum				PCM			
structure	E, (kcal/mol)	ϕ(FSNS), °	ϕ(SNSC), °	ϕ(NSCC), °	E, (kcal/mol)	ϕ(FSNS), °	ϕ(SNSC), °	ϕ(NSCC), °
ggc	0.00	76.6	83.1	33.2	0.00	77.4	83.4	32.7
ggg	0.22	76.9	74.8	137.9	0.33	76.3	76.0	139.3
ggt	0.42	76.1	93.9	-170.0	0.40	75.3	95.0	-171.7
ggg’	0.56	76.0	100.3	-62.5	0.67	75.8	101.6	-62.6

Table S4. Partial Atomic Charges Calculated from the Fit to the Electrostatic Potential at the MP2/aug-cc-pVDZ Level in Vacuum.

TFSI

atom	*trans*	*g1*	*g2*	average
O	-0.458224	-0.461139	-0.450985	-0.45678
S	0.776102	0.85154	0.832019	0.81989
N	-0.538847	-0.558279	-0.539889	-0.54567
S	0.77704	0.882158	0.76469	0.80796
O	-0.480048	-0.47674	-0.484384	-0.48039
C	0.416878	0.294014	0.290694	0.33386
O	-0.479827	-0.49709	-0.452234	-0.47638
C	0.415944	0.26006	0.311097	0.32903
O	-0.458486	-0.491385	-0.458994	-0.46962
F	-0.187867	-0.160446	-0.10991	-0.15274
F	-0.133562	-0.114966	-0.134675	-0.12773
F	-0.164157	-0.135117	-0.159531	-0.15294
F	-0.187678	-0.145467	-0.14769	-0.16028
F	-0.133411	-0.125261	-0.128684	-0.12912
F	-0.163856	-0.121883	-0.131524	-0.13909
average C	0.41641	0.27704	0.30090	0.33145
average S	0.77657	0.86685	0.79835	0.81392
average O	-0.46915	-0.48159	-0.46165	-0.47079
average F	-0.16176	-0.13386	-0.13534	-0.14365

FPFSI

atom	*ggc*	*ggg*	*ggt*	*ggg'*	average
C (F2)	0.126267	0.032488	0.156071	0.110928	0.10644
F 2	-0.145391	-0.090429	-0.137109	-0.129798	-0.12568
S (SO_2)	0.840805	0.835478	0.811209	0.801207	0.82217
O (SO_2)	-0.448607	-0.47143	-0.473593	-0.457025	-0.46266
O (SO_2)	-0.479212	-0.449632	-0.443192	-0.438308	-0.45259
N	-0.515145	-0.505849	-0.500557	-0.505529	-0.50677
S (SO_2F)	0.879077	0.880007	0.883757	0.872117	0.87874
O (SO_2F)	-0.452105	-0.436621	-0.438606	-0.433115	-0.44011
O (SO_2F)	-0.441816	-0.453406	-0.459427	-0.453524	-0.45204
F 1	-0.244058	-0.242067	-0.246227	-0.242013	-0.24359
C (F3)	0.422413	0.407452	0.400219	0.33811	0.39205
F 2	-0.101417	-0.073454	-0.108643	-0.108024	-0.09788
F 3	-0.173353	-0.166673	-0.165943	-0.157536	-0.16588
F 3	-0.135778	-0.12537	-0.142802	-0.092093	-0.12401
F 3	-0.131681	-0.140495	-0.135159	-0.105396	-0.12818
average O (SO_2)	-0.46391	-0.46053	-0.45839	-0.44767	-0.45762
average O (SO_2F)	-0.44696	-0.44501	-0.44902	-0.44332	-0.44608
average F2	-0.12340	-0.08194	-0.12288	-0.11891	-0.11178
average F2	-0.14694	-0.14418	-0.14797	-0.11834	-0.13936

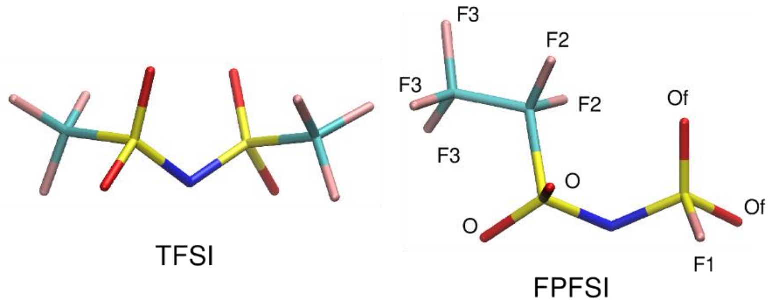

Figure S1. Structures of TFSI and FPFSI anions and the labeling of atoms in FPFSI.

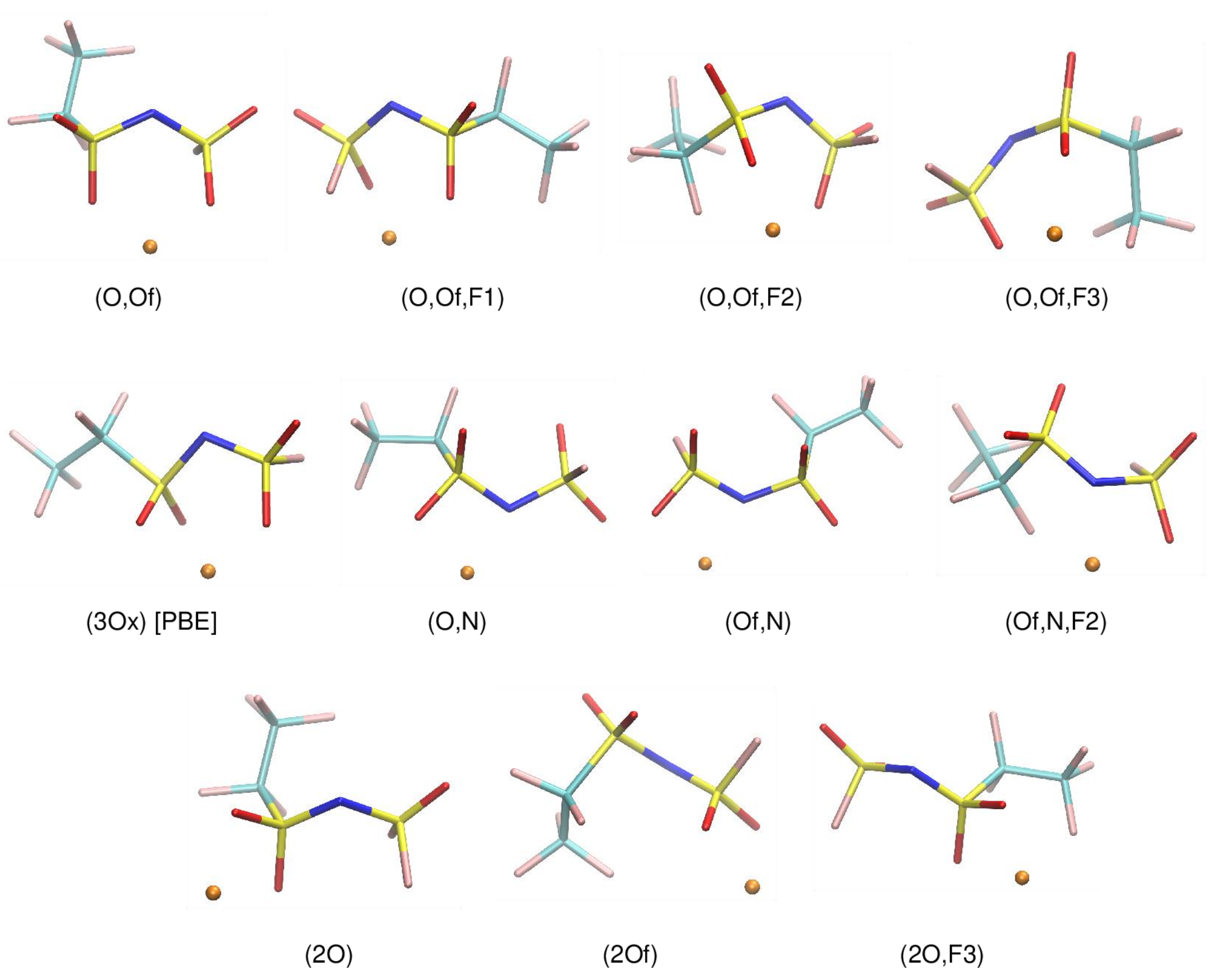

Figure S2. Geometries of the most stable LiFPFSI structures of each type obtained at the MP2/aug-cc-pVDZ level in vacuum. The 3Ox geometry was taken from the PBE calculations.

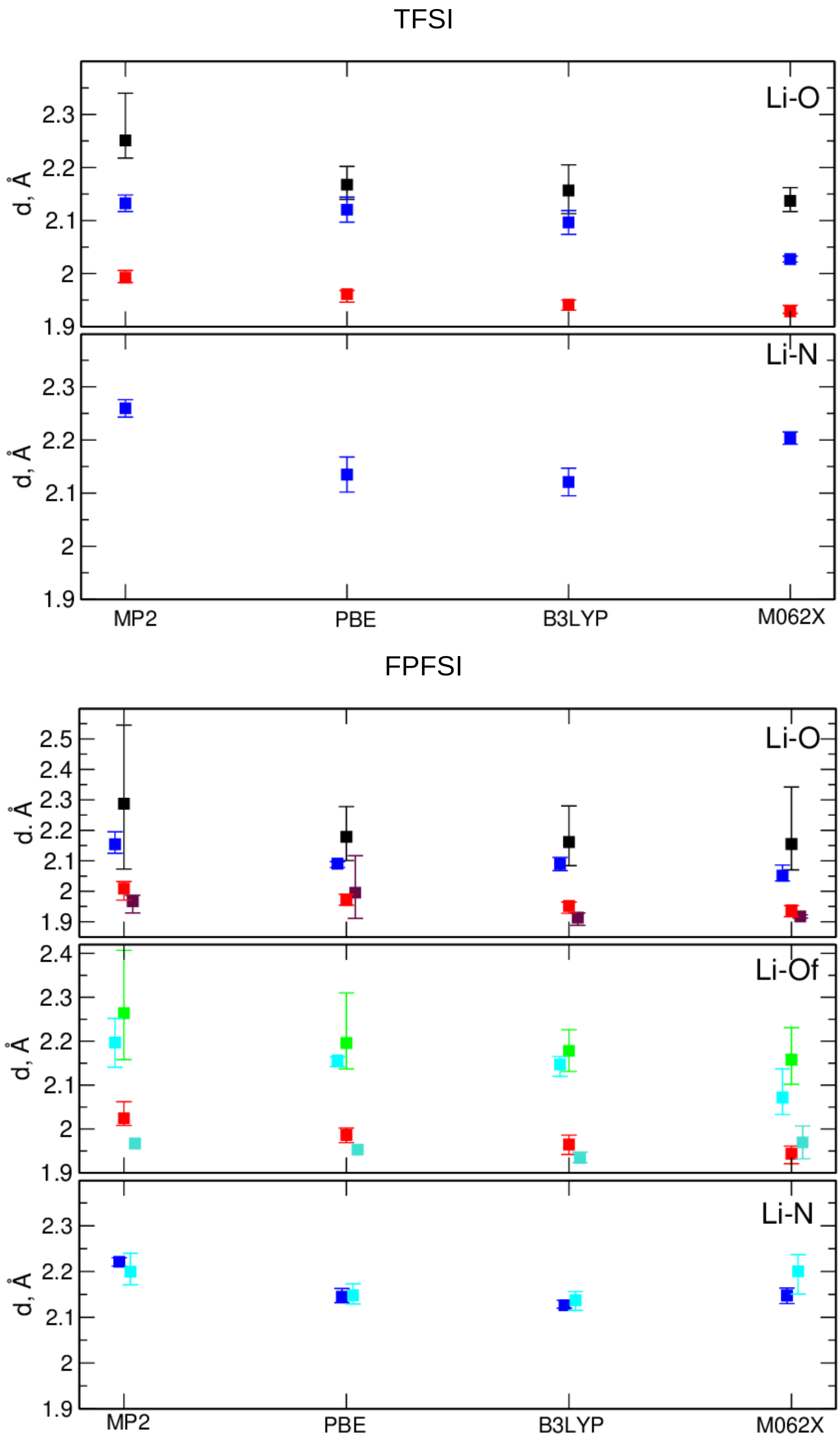

Figure S3. Li-anion distances obtained in the PCM calculations. Points mark the average value. Colors label the type of coordination according to Figures 4 and 6 in the main paper.

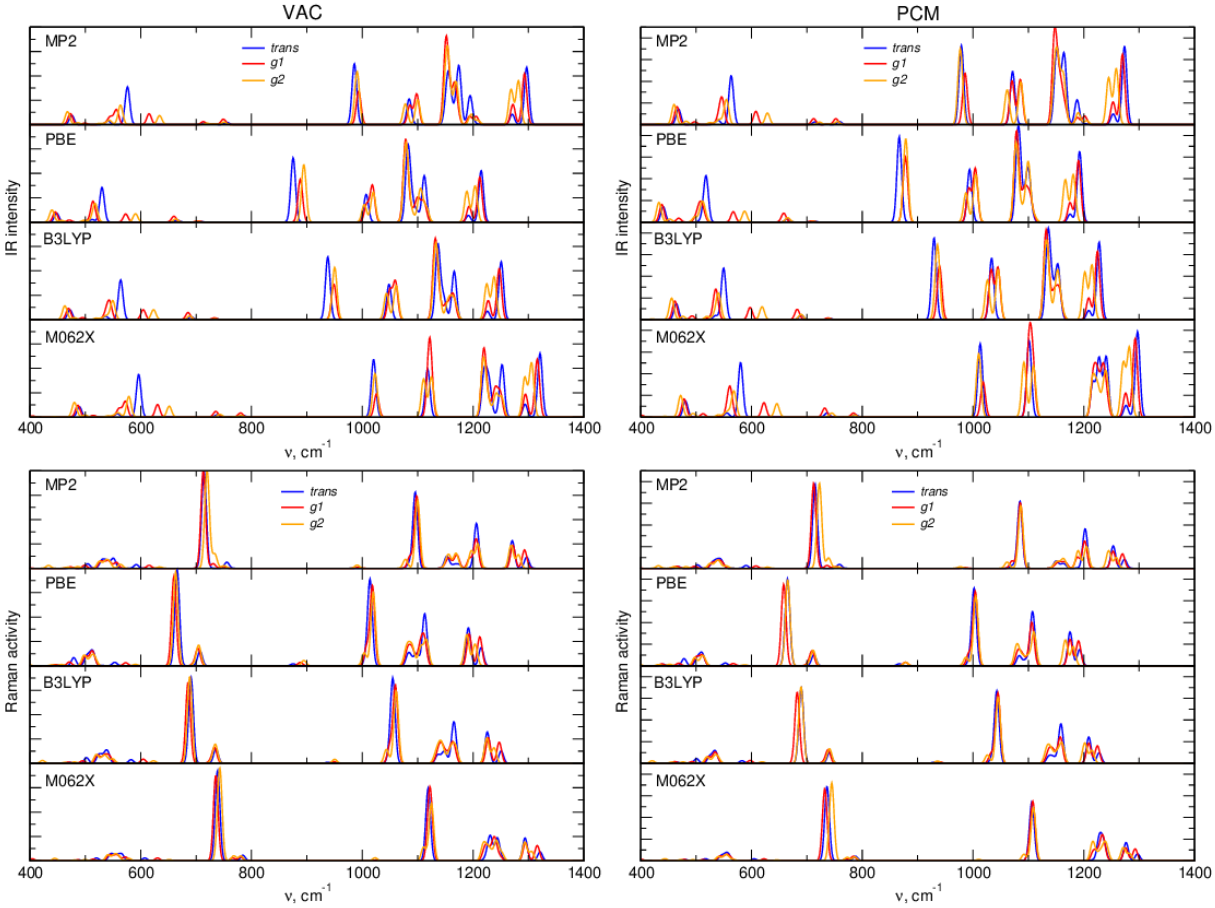

Figure S4. Vibrational spectra of TFSI anion obtained within different methodologies.

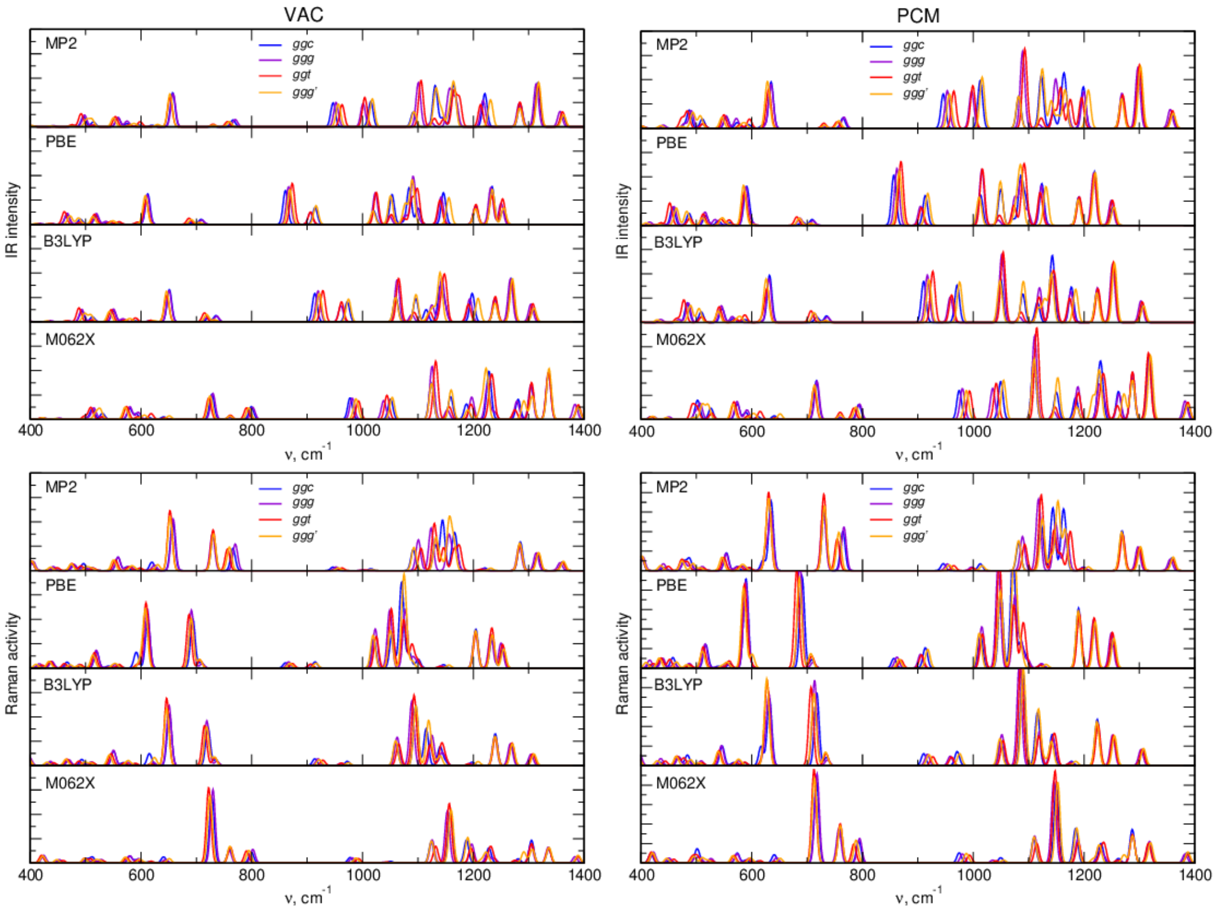

Figure S5. Vibrational spectra of FPFSI anion obtained within different methodologies.

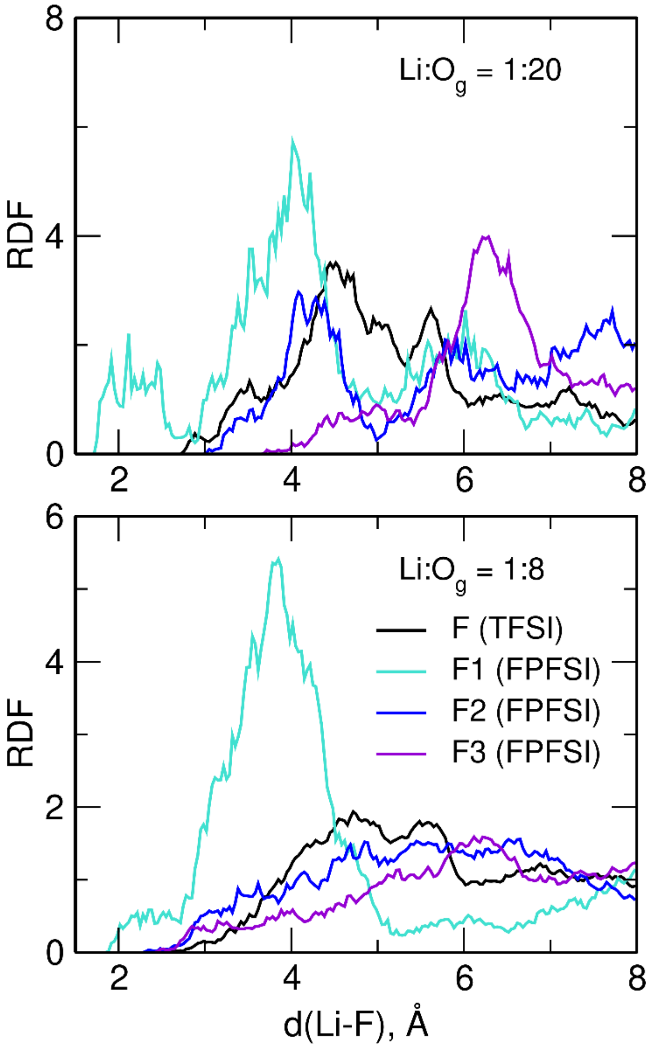

Figure S6. Li-F RDFs from the AIMD simulations for LiTFSI and LiFPFSI electrolytes of type *b*.

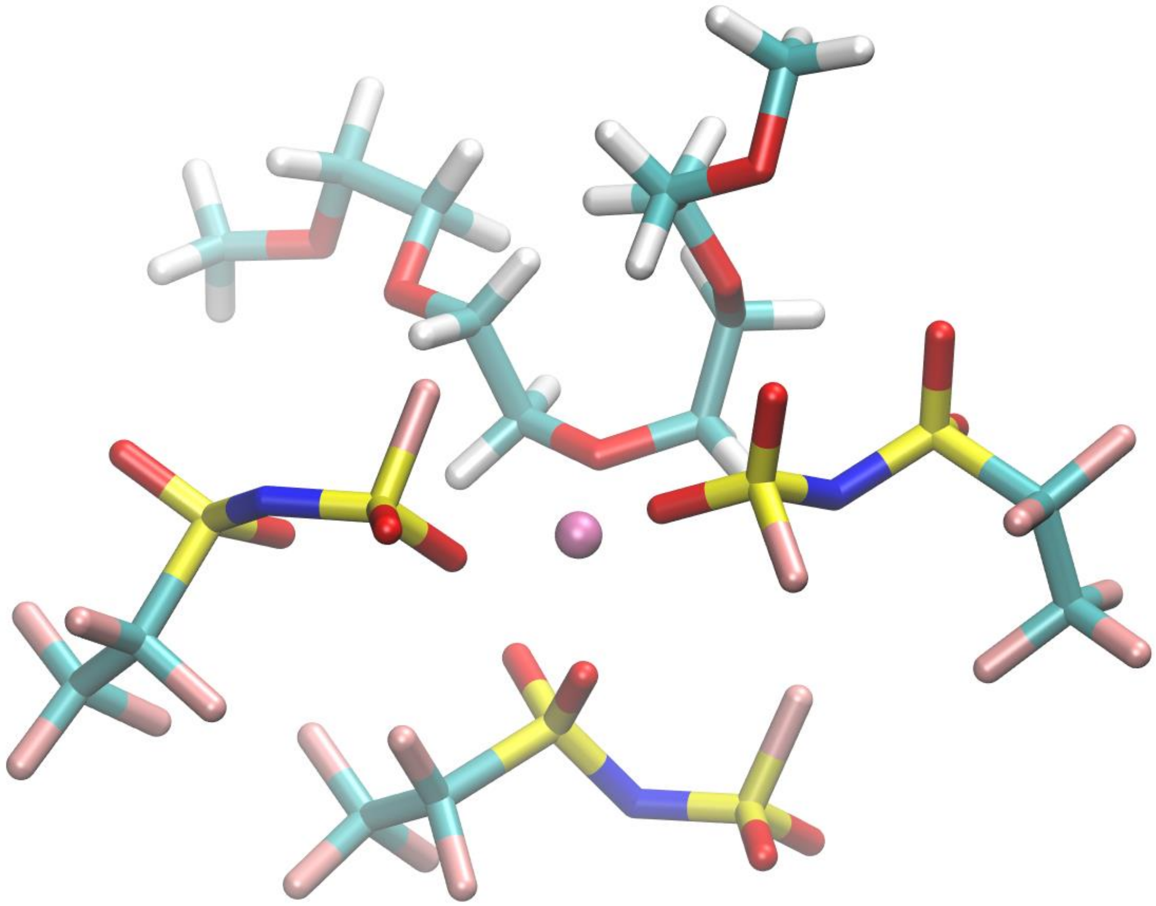

Figure S7. A sample snapshot of the 1:8 LiFPFSI-*b* electrolyte showing the Li^+ coordination to three FPFSI anions.

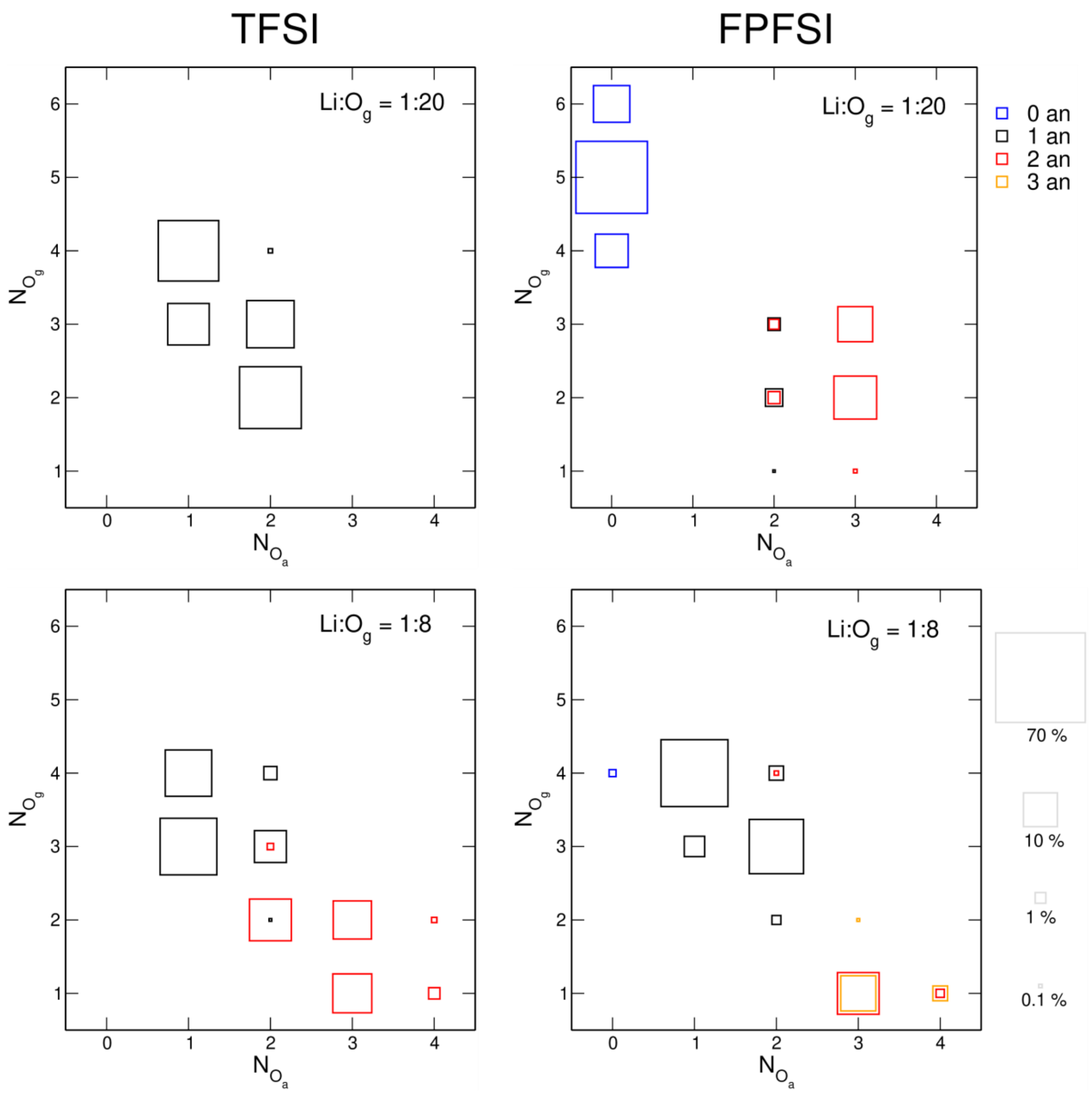

Figure S8. Probabilities of different coordination environments in the electrolytes of type *b*. Areas of the squares are proportional to the probability.

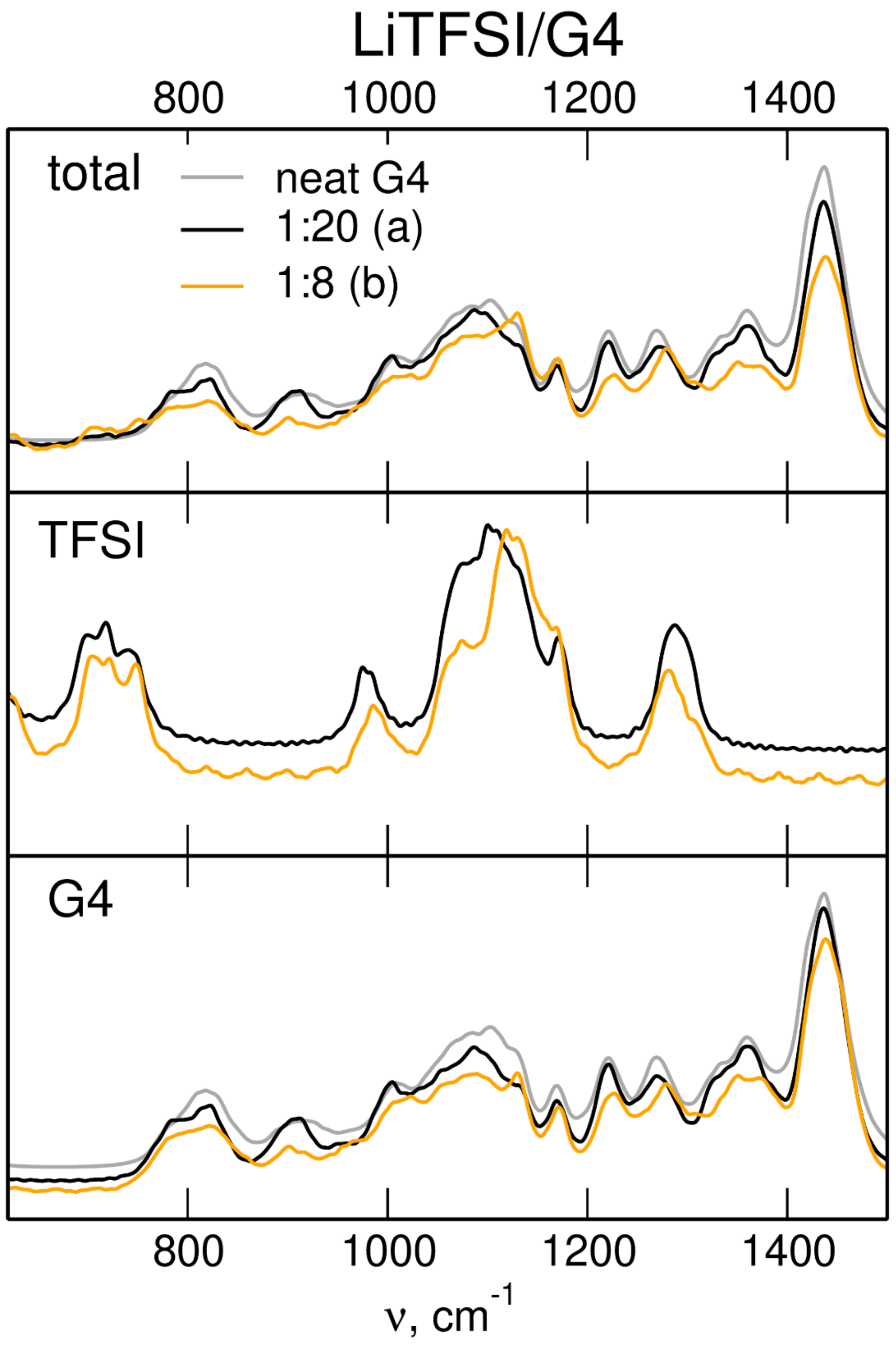

Figure S9. Power spectra (Fourier transforms of velocity autocorrelation function) for neat G4 and LiTFSI/G4 electrolytes: total (upper panel); for anions (middle panel); and for tetraglyme (bottom panel). Colors label different electrolytes.

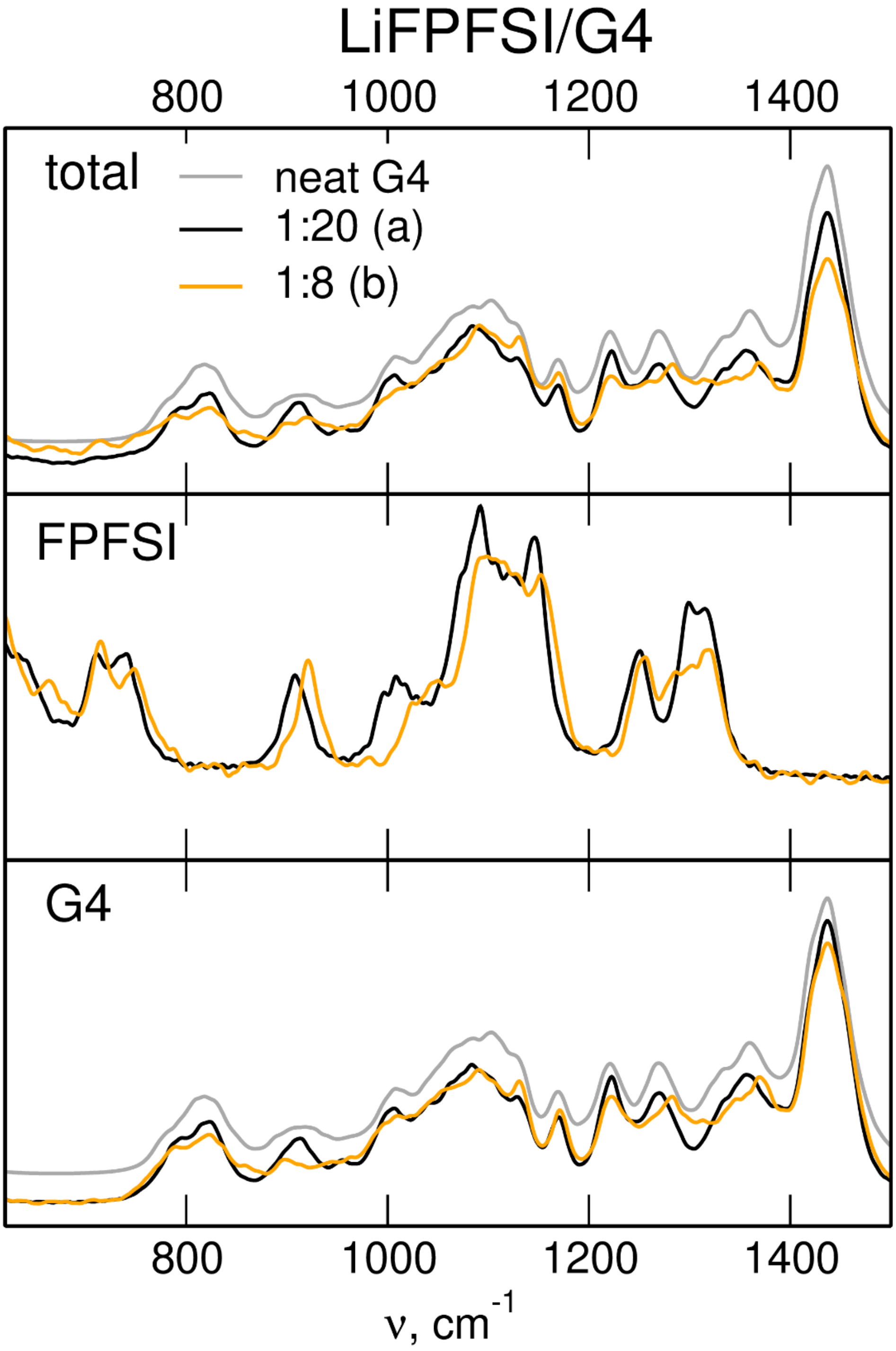

Figure S10. Power spectra (Fourier transforms of velocity autocorrelation function) for neat G4 and LiFPFSI/G4 electrolytes: total (upper panel); for anions (middle panel); and for tetraglyme (bottom panel). Colors label different electrolytes.

Appendix: MP2-optimized structures of *gauche* TFSI conformers

structure ***g1***

G09 route section:

```
# mp2/aug-cc-pvdz opt=Tight freq
```

Final MP2 energy: -1824.1155281641 a.u.

Low frequencies --- -0.1640 -0.0905 -0.0033 -0.0020 -0.0014 0.3200
Low frequencies --- 35.0494 46.3806 51.9243

Geometry convergence:

```
         Item               Value     Threshold  Converged?
 Maximum Force            0.000001     0.000015     YES
 RMS     Force            0.000000     0.000010     YES
 Maximum Displacement     0.000037     0.000060     YES
 RMS     Displacement     0.000010     0.000040     YES
```

final coordinates (Å):

```
 O    -0.197105     1.887343    -0.560815
 S    -1.082784     0.823269    -0.017359
 N    -0.354213    -0.427700     0.735057
 S     1.135004    -0.933247     0.266153
 O     1.668174    -0.421001    -1.025750
 C    -1.876448     0.074094    -1.546342
 O    -2.267699     1.188271     0.806007
 C     0.701021    -2.721540    -0.089557
 O     2.038183    -1.037152     1.445109
 F    -2.656727     1.005958    -2.149405
 F    -0.960035    -0.345452    -2.439257
 F    -2.662527    -0.974873    -1.213765
 F     1.821985    -3.389257    -0.459545
 F     0.189376    -3.336023     0.997943
 F    -0.194171    -2.818227    -1.095826
```

structure ***g2***

G09 route section:

```
# mp2/aug-cc-pvdz opt=Tight freq
```

Final MP2 energy: -1824.1152739079 a.u.

Low frequencies --- -0.1254 -0.0844 -0.0025 0.0012 0.0017 0.1058
Low frequencies --- 18.1291 36.0879 49.0414

Geometry convergence:

```
         Item               Value     Threshold  Converged?
 Maximum Force            0.000001     0.000015     YES
 RMS     Force            0.000000     0.000010     YES
 Maximum Displacement     0.000052     0.000060     YES
 RMS     Displacement     0.000020     0.000040     YES
```

final coordinates (Å):

```
 C    -2.293811    -0.747422    -0.534252
 F    -1.789851    -0.435931    -1.742033
 F    -2.176255    -2.083070    -0.352504
 S    -1.435854     0.190938     0.844967
 N     0.082309    -0.389883     0.791138
 S     1.203697     0.494112    -0.026877
 O     1.816193     1.596080     0.767549
 F    -3.616537    -0.441962    -0.535169
 O    -2.111984    -0.343491     2.057509
 O    -1.598882     1.621775     0.460285
 O     0.937702     0.723649    -1.476177
 C     2.487439    -0.870432     0.002432
 F     3.603259    -0.412656    -0.618234
 F     2.825332    -1.223419     1.259380
 F     2.066679    -1.970503    -0.655392
```
